# Phenotypic Examination of *Camelina sativa* (L.) Crantz Accessions from the USDA-ARS National Genetics Resource Program

**DOI:** 10.3390/plants9050642

**Published:** 2020-05-19

**Authors:** Sara K. Hotton, Meridith Kammerzell, Ron Chan, Bryan T. Hernandez, Hugh A. Young, Christian Tobias, Thomas McKeon, Jenny Brichta, Nathan J. Thomson, James G. Thomson

**Affiliations:** 1 Bayer Crop Science, West Sacramento, CA 95605, USA; sara.hotton@bayer.com; 2 Cargill, Inc., Fort Collins, CO 80525, USA; Meridith_Kammerzell@cargill.com; 3 Crop Improvement and Genetics, USDA-ARS-WRRC, Albany, CA 94710, USA; ronald.chan@usda.gov (R.C.); christian.tobias@usda.gov (C.T.); Thomas.McKeon@USDA.GOV (T.M.); jenny.brichta@usda.gov (J.B.); 4 Department of Plant Sciences, University of California, Davis, CA 95616, USA; Rhernandez@sfwater.org; 5 Novozyme, Franklinton, NC 27525, USA; HYO@novozymes.com; 6 Blinn College, Bryan, TX 77833, USA; Nathan.Thomson31@buc.blinn.edu

**Keywords:** *Camelina sativa*, oil seed crop, National Genetic Resources Program (NGRP), agronomic traits, biotechnology

## Abstract

*Camelina sativa* (L.) Crntz. is a hardy self-pollinated oilseed plant that belongs to the *Brassicaceae* family; widely grown throughout the northern hemisphere until the 1940s for production of vegetable oil but was later displaced by higher-yielding rapeseed and sunflower crops. However, interest in camelina as an alternative oil source has been renewed due to its high oil content that is rich in polyunsaturated fatty acids, antioxidants as well as its ability to grow on marginal lands with minimal requirements. For this reason, our group decided to screen the existing (2011) National Genetic Resources Program (NGRP) center collection of camelina for its genetic diversity and provide a phenotypic evaluation of the cultivars available. Properties evaluated include seed and oil traits, developmental and mature morphologies, as well as chromosome content. Selectable marker genes were also evaluated for potential use in biotech manipulation. Data is provided in a raw uncompiled format to allow other researchers to analyze the unbiased information for their own studies. Our evaluation has determined that the NGRP collection has a wide range of genetic potential for both breeding and biotechnological manipulation purposes. Accessions were identified within the NGRP collection that appear to have desirable seed harvest weight (5.06 g/plant) and oil content (44.1%). Other cultivars were identified as having fatty acid characteristics that may be suitable for meal and/or food use, such as low (<2%) erucic acid content, which is often considered for healthy consumption and ranged from a high of 4.79% to a low of 1.83%. Descriptive statistics are provided for a breadth of traits from 41 accessions, as well as raw data, and key seed traits are further explored. Data presented is available for public use.

## 1. Introduction

Renewable energy sources including esterified vegetable oil (i.e., biodiesel) have been proposed as a possible option to reduce greenhouse gas (GHG) emissions in the transportation sector. Current widely used oilseed for producing biofuel include rapeseed, sunflower (Europe), soybean (USA), and palm oils (tropical regions). In comparison to these other oilseed plants, *Camelina sativa* (L.) Crntz. (camelina) has a very short life cycle (52 to 72 days) and is economical to grow on marginal lands due to minimal nutritional input requirements. It further has the capacity for both summer and winter crop production. The plant is native to northern Europe and Southeast Asia and is a member of the mustard family (Brassicaceae). This ancient crop has previously been used for cooking, cosmetics, and fuel oil [1]. Archaeological studies date its cultivation back to the Bronze Age [2,3]. It was utilized throughout the northern hemisphere, until the 20th century when it was replaced by the high yielding crops, canola, rape, and soy [4,5]. Despite its potential, there is a limitation to more widespread use due to the lack of agronomic knowledge, as well as limited information about the genetic diversity in the available germplasm. 

Currently, the crops rapeseed, sunflower, soybean, and palm are primarily used for food and their use in generating oil for biodiesel competes directly with their value as food. Camelina appears an ideal alternative crop for biodiesel as it would not compete with current food applications, contains high oil content that is rich in polyunsaturated fatty acids and tocopherols, which confers stability against oxidation. Due to its oil composition, camelina can be a valuable renewable resource for the production of biodiesel, hydraulic oil, lubricants, and jet fuel [6,7,8,9]. 

Among non-food plants, camelina is well suited for temperate climates with poor soils, thus can be grown on marginal lands. Its beneficial agronomic and economic attributes include good yield under drought conditions, low fertilizer requirement, pesticide demand, compatibility with existing agricultural equipment and short growth time [6,7,8,9]. Camelina also displays allelopathic characteristics, discouraging the growth of weeds [10]. With its allelopathic nature, early germination characteristic, and its ability to be sown under freezing conditions, camelina makes an excellent cover-crop. These useful agronomic traits and the fact that camelina has been shown to surpass yields of oilseed crops such as flax under drought-like conditions make it an appealing crop for production in the inland Pacific Northwest and North American semi-arid prairies.

Additionally, the by-product from oil collection provides a meal that is rich in protein and vitamin E, which in turn has prompted studies for its use as an aquaculture and animal feed supplement [11,12,13,14,15,16,17,18,19]. The meal has been shown to be composed of 45% protein, 13% fiber, 5% vitamins and minerals, as well as 10% of oil residue [20]. The protein composition includes the amino acids glutamine, asparagine, arginine, leucine, glycine, valine, serine, lysine, and proline in a ratio comparable to rapeseed and soy. However, studies have shown that use of camelina meal in many cases alters the tissue fatty acid composition. It further contains glucosinolates and phytates and therefore must be consumed at low levels. As such, the meal collected from crushed seeds is used as a limited supplement for animal feed [21,22] with poultry and broiler chicken feed rations at a maximum of 10%, beef cattle rations up to 10%, swine feed rations are set at 2%, while Canadian aquaculture is allowed a 3% ration [23]. However, breeding and or genetic modification offers avenues to improve both the oil and meal composition to meet growing needs. 

While the majority to nutritional surveys have been conducted for animal feed there are studies suggesting that camelina oil added to a persons’ diet could improve their serum lipid profile [24,25] offering an avenue for its eventual use in the human food stream. However, certain considerations such as low (<2%) erucic acid levels [6,26] would need to be addressed by screening, breeding, or biotechnology before widespread utilization occurred. Alternatively, as camelina oil is a rich source of linolenic acid and omega tocopherols, it has potential use in both nutraceuticals and cosmetics [27].

Camelina offers an array of benefits to both the producer and the consumer, therefore exploring untapped genetic reserves appears to be the next logical step. To date, a limited number of camelina lines have been used in field trials, or for biotechnological improvement. Even though there are a large number of cultivars in existence, with tremendous genetic potential, these cultivars have been poorly characterized, and many quality characteristics have not been evaluated at all [28]. Examples of this can be seen in the variation in camelina seed oil content that has been reported to range between 320 and 460 g kg^−1^, and linolenic acid concentrations ranging from 28 to 43%. Results with large seeded genotypes have shown there exists an inverse correlation between 1000 seed weight and oil content and therefore these cultivars are considered inferior to small seeded cultivars as an oil source [7,29]. Variability also exists for yield with reported ranges from 0.336 t ha^−1^ to 2.25 t ha^−1^ depending on genotype and location grown [7,30,31]. Although, these results could be and are most likely due to environmental factors as well as genetic, more studies will be needed using true breeding populations of camelina with a large set of known QTLs to determine.

Currently, camelina stands on the brink of multiple industrial applications and potential animal feed use. Above and beyond its apparent native potential is the fact that this species can be genetically manipulated with relative ease [8,23,32,33,34,35,36,37,38]. Published results of a selection of germplasm treated to a mutagenesis technique has led to camelina cultivars with higher oil content and improved fatty acid composition, such as the cultivar Blaine Creek that is enriched in *ω*-3 fatty acids, and Suneson, which has shown 2–3% higher oil content with enhanced *α*-linolenic acid [39,40].

Another option is the use of direct genomic modification utilizing biotechnological techniques. Being a close relative of Arabidopsis, there are 30 years of molecular biology techniques for genetic modification available for use [41,42,43]. To date, genome modification has been used to enhance the metabolic pathway for fatty acid production in order to enhance production levels [32,33,44,45,46,47]. Transcriptome analysis and comparative genomics have provided a wealth of information for both breeders and biotechnological applications [48,49,50,51,52,53,54]. Sophisticated molecular techniques for genome modification such as RNAi [21] and CRISPR [55] have been utilized for reducing or abolishing gene expression to shift the fatty acid metabolic process. While techniques such as recombinase mediated cassette exchange (RMCE) [56] or gene assembly in agrobacterium by nucleic acid transfer using recombinase technology (GAANTRY) [57] could be employed for addition of large genetic pathways for novel metabolic engineering projects. These modifications could be used to tailor camelina oil or meal for specific uses [58,59,60,61,62,63]. An end goal would be, for these plant-based oils, to become an option to replace non-sustainable petroleum-based products as fuel, lubricants and specialty chemicals. For a review of biotechnological improvements to Camelina, see Bansal et al. [64].

However, even with the advances seen in molecular modification certain basic biotechnological tools are still lacking, such as selectable markers genes. To date the herbicide bar selection system is the only robust selection method available. Others marker genes such as hygromycin (*hptII*) [43] and acetolactate synthase (*ALS*) [42,63,64,65] have been published, but do not appear as robust as bar, and therefore may have limited use as a biotechnological tool.

Genetic and other background information about the origin of particular camelina genotypes is essential for initiating focused breeding programs [66] but is not available or cross referenced for most of the available National Genetic Resources Program (NGRP) accessions. The aim of the present study has been to provide an overview of the phenotypic diversity present within the NGRP camelina germplasm collection, as of 2011. We attempt to capture and present agronomically important traits and collate this information with accessible and searchable NGRP camelina accessions. Data is presented on 41 cultivars that include traits such as the range of germination rates, time to bolt, heritable seed traits—such as seed size, 1000-seed weight, oil content, fatty acid—as well as chromosome counts. We further provide information on the use of novel selection marker genes for potential utilization in biotechnological applications. Data is presented in an uncompressed format in both tables for manuscript discussion and as raw data in XLS spreadsheet (Supplemental Tables S1–S4) format. 

## 2. Results

Identifying camelina plant and seed quality traits for important agronomic characteristics for marketing and processing is needed to develop this crop which is in direct competition with other oilseeds currently in production. Therefore, we measured a number of traits that include 1000-seed weight, oil content, and performed compositional analysis. Agronomic characteristics were also monitored for the different genotypes under controlled conditions in order to minimize environmental factors and evaluate genetic influences and to catalogue phenotypic responses. Our lab initially obtained 38 camelina accessions from the NGRP for evaluation but four of the lines (PI 597833, PI 650142, PI 650157, PI 650158) were discovered to have mixed seed from two distinct cultivars. These lines were therefore given designations of ‘A’ and ‘B’ types of the originating name. The NGRP collection cultivar PI 304268 appeared to require vernalization conditions that were not met in this study, as such, the plant remained in the rosette stage, failing to flower and provide seed. Therefore, this line was not included in this study; giving a total of 41 accessions reviewed.

### 2.1. Plant Description

Camelina is a self-pollinated plant with small, 4-lobed flowers of pale yellow. The tear shaped fruits, or siliques, appear similar to flax bolls and contain approximately 15–20 small seeds with a high oil content, making it desirable for potential commercialization. Throughout the study, phenotypic descriptions and growth stages of camelina sativa presented will be subtitled according to the two-digit BBCH scale [67] in the text. 

### 2.2. Early Development

Principal growth stage, seed germination, and early development. 01: Initiation of seed imbibition—It was observed that all viable seed for every cultivar formed a mucosal/gelatinous coat within the first 5 min of being imbibed. 03: Radicle emergence from seed—All cultivars showed radical emergence in 1 to 2 days with a median rate of 1 day (Table 1). 04: Emergence of hypocotyl with cotyledons from the seed—Shoot emergence was seen from 1.8 to 3 days after sowing on damp soil and with a median rate of 2 days (Table 1). At day 4, cultivars were measured for root length, hypocotyl length, and percent cotyledon unfolding. Roots were found to range from 5.4 mm to 40.4 mm with a median value of 25.9 mm (Table 1). Hypocotyl length was determined to have values from 2.2 mm to 10.8 mm with a median value of 7.8 mm (Table 1). 10: Cotyledons (node 0) unfolded—The rate of cotyledon unfolding was measured at day 4 and was observed to range from 0 to 100% with a median value of 72%. Vernalization was required for 9 of the 41 cultivars and these are indicated by asterisks at the end of their accession number (Table 1).

### 2.3. Physical Attributes

**Figure 1:**
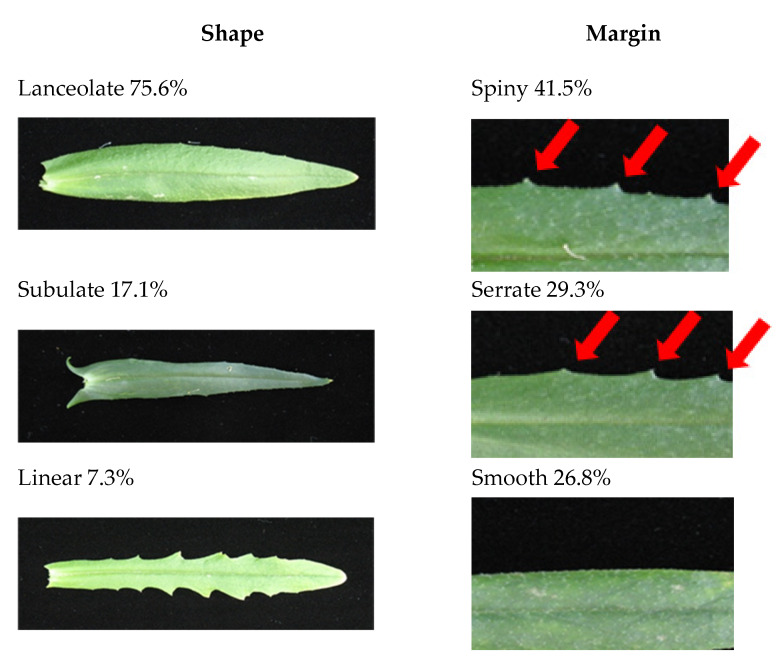
Leaf shape and margin. Shape and margin of the leaf were determined after plants had bolted and reached mature height. Lanceolate (narrow oval shape tapering to a point at each end) was seen in 75.6% of population. Subulate (slender and tapering to a point) was observed 17.1% and Linear (long and very narrow like a blade of grass) seen 7.3%. The Spiny (stiff, sharp points such as thistles) margin was most prevalent in the population at 41.5%. Serrated (saw-toothed; with asymmetrical teeth pointing forward) was seen 29.3% and Smooth (even margin without points) was 26.8%. Points are small needle like projections seen at the leaf edge seen in the spiny and serrated examples with red arrows above.

Principal growth stage 1: leaf development. Leaf morphology was observed in three basic shapes with lanceolate (75.6%) being the most predominant, followed by subulate (17.1%) and linear (7.3%) (Figure 1, Table 2). Leaf margin morphology (shape of the edge) was determined to also have three basic characteristics with spiny (41.5%) most often observed. Serrate (29.3%) was the second most common followed by smooth (26.8%). Examples of shapes are seen in Figure 1 and each accession’s combination of leaf shape, edge shape, and spine number (points) characteristics are listed in Table 2. Like most of the Brassicaceae, camelina develops lateral branches. The development of lateral branches is variable and depends on the genotype. Plant density and environmental conditions that may also affect the number of branches [67]. Here we present findings of lateral branch development under low plant density and favorable growth (greenhouse) conditions.

**Figure 2:**
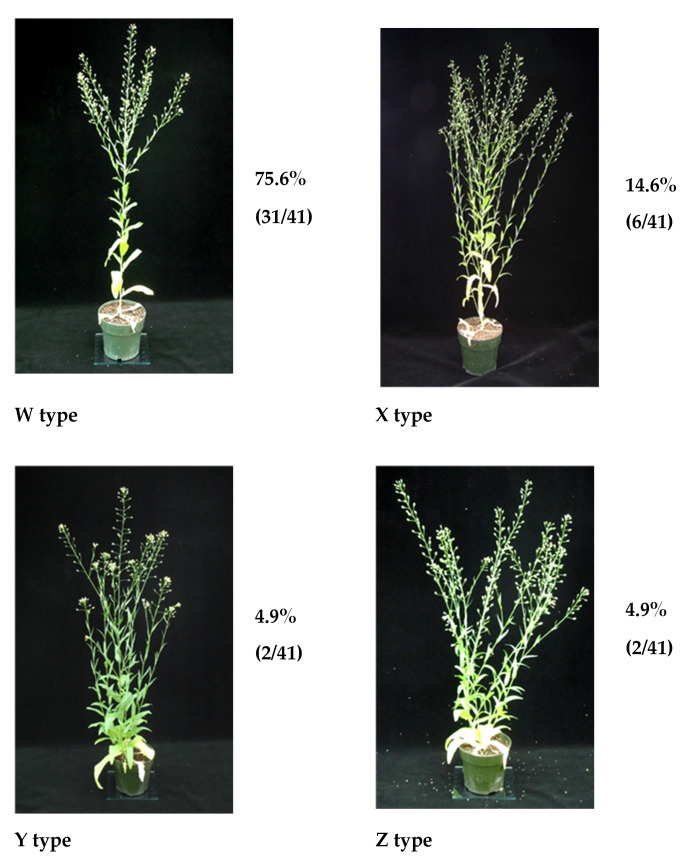
Branch types. W type—branched only at top and seen in 75.6% of population. Cultivar PI 597833B shown. X type—branched down length of main stem. Observed in 14.6% of population; PI 25366 depicted. Y type—tiller like; most all branches originating from the base. Seen in 4.9% of the population; PI 311736 pictured. Z type—chaos; no main stem observed and secondary branches emerging everywhere. Observed in 4.9% of cultivars; PI 650168 shown as an example.

Lateral branch development appears to have four unique patterns. Top heavy branching (W—75.6%) often seen with secondary branching is by far the most common, followed by branched length of main stem (X—14.6%) with few secondary branches observed, Tiller (Y—4.9%) where all branches originate from the base and contain few secondary branching and finally Chaos (Z—4.9%) where branches are seen originating everywhere without a dominant stem observed (Figure 2, Table 2). Interestingly, four cultivars PI 311736, PI 650143, PI 650152, and PI 650167 appeared to keep the rosette throughout their lifecycle, under greenhouse conditions. All appear to be winter cultivars. 

The cultivars in this study showed a range of bolting time from 18 to 35 days with an average of 23 days (Table 3). Principal growth stage 6: flowering (main shoot); 65: Full flowering: 50% of flowers open—The primary inflorescence is a composite flower composed of the number of florets and defined as ‘open’ when greater than 50% of the florets had unfolded. This occurred in a minimum of 29 days and a maximum of 43 days with the median being 33 days (Table 3). The number of leaves present on the primary bolting stem at time of flowering ranged from 18 to 41 (Table 3). The mean value height and width (Table 3) seen at the time of initial flowering were 45 cm and 22 cm, respectively. The primary inflorescence contains 9 florets at a minimum and 18 at a maximum with a median value of 12, while the secondary inflorescence contains an average of 4 florets (Table 3).

Maturity of the plant was defined as the time when the plant is in full flower, it has reached its maximum height and seed set has begun. The earliest accessions to reach maturity were PI 650164 and PI 650165 at 42 days and the latest at 63 day was seen by PI 650143. A median maturity value of 50 days was observed (Table 4). 39: Maximum stem length—Accessions reached maximum height at maturity, which was seen to be between 64.9 cm (PI 650142A) to a maximum of 88.3 cm (PI 650151) with a median value of 78.3 cm (Table 4). The plants width at maturity was observed to be between 11.4 cm (PI 650153) to 61.0 cm (PI 650168) with a median value of 36.6 cm (Table 4). Principal growth stage 9: senescence. 97: Plant dead and dry—Plants were dry and seeds ready to harvest at a minimum of 52 days (PI 304271, PI 650164, PI 650165), at a maximum of 72 days (PI 650145, PI 650146) with an mean value of 63 days (Table 4). 

### 2.4. Seed Analysis

Seed characteristics were assessed, and the mean seed weight was seen to range from 0.19 to 1.05 mg per seed (Table 5). Total mean seed weight per plant showed a minimum of 1.45 g (PI 304270) to a maximum of 5.06 g (PI 311735) per plant with a median value of 2.6 g (Table 5). The estimate total seed per plant shows a range from 1604 (PI 650153) to 9225 (PI 650143) seed per plant with a median value of 3328 (Table 5). While the number of seed and total weight of seed per plant did not appear to correlate, the ‘winter’ cultivars produced greater than average amounts of seed in general. Seed dimensions showed a mean minimum and maximum width of 0.65 and 1.06 mm, respectively (Table 5). The seed mean length ranged from 1.20 to 2.12 mm with an average of 1.80 mm (Table 5). The length by width ratio was also compiled and 1.74 to 2.62 and a median value of 2.04 (Table 5). 

### 2.5. Seed Oil Biochemical Data

Biochemical analysis of the oil collected shows varying degrees of differences among the 41 accessions of *Camelina sativa* (L.) Crntz. (Table 6). Oil content (OC) ranged from 23.6% in PI 650152 to 44.1% in the PI 650155 accession (Table 6). Fatty acid components included saturated, monounsaturated, and polyunsaturated fatty acids. The most prevalent saturated fatty acid was palmitic acid, ranging from 5.5 % to 9.5 % (Table 6). The most abundant mono-unsaturated fatty acids are oleic acid (C18:1), ranging from 9.1% to 17.1%, and gondoic acid (C20:1), measured at 10.5% to 16.4% (Table 6). The most abundant poly-unsaturated fatty acids are linoleic acid (C18:2), from 16.1% to 28.6%, and linolenic acid (C18:3), measured at 23.5% to 36.2% (Table 6). The weight of a thousand seeds (TWS) varied from 0.2006 g in PI 650167 to 1.0473 g PI 650153 (Table 6). See Supplemental Table S1 for a complete fatty acid profile of the raw data obtained. To further explore the oilseed yield traits of thousand seed weight (TSW) and total seed weight, one-way ANOVAs were performed to compare accessions. Significant differences between accessions were observed for both TSW (ANOVA, *p* < 2.2 × e^−16^) and total seed weight (ANOVA, *p* < 2.2 × e^−16^). TukeyHSD post-hoc analyses were performed for all pair-wise comparisons with results included in Supplemental Table S3 (TSW stats) and Supplemental Table S4 (total seed weight stats).

### 2.6. Genetic Analysis

The camelina accessions were examined for chromosome number. It was determined that 75.6% of the lines contained the expected 2*n* = 40 while unexpectantly 24.3% contained a 2*n* = 38 value. One accession PI 650152 contains a 2*n* of 26. Individual lines were also examined for their COT values to provide a unique identifying fingerprint for each cultivar (Table 7). The COT analysis is a technique that provides a way to measure DNA reassociation kinetics and gives a measure of repetitive DNA content per genome [68]. This analysis was used as verification for the predicted A/B split in the 4 lines that contained observable differences in phenotypes, and independent from chromosome counts. Table 8 provides a summary of previous tables but specific to the alignment of the A/B accessions. Results show that each A/B accession has a unique COT value, chromosome number, vernalization requirement, branch pattern, leaf shape, leaf margin, flowering time, height, and number of days to maturity. 

### 2.7. Advances in Biotechnology

Biotechnological enhancements have been achieved to complement the traditional cultivar improvement efforts of breeding and mutagenesis. To add to and improve upon these biotechnology efforts, our lab investigated the use of positive as well as negative selection marker genes to facilitate techniques such as RMCE and GAANTRY [56,57] for metabolic engineering. 

We chose to investigate the positive marker genes bar, [41], *hptII* [69], *nptII* [70] and *sulI* [71] for use in camelina selection, as they have all been shown to work in Arabidopsis. The optimal range of selective agents to inhibit camelina growth was determined for each (Supplemental Figures S1–S5). The negative selective marker gene, *codA,* was also investigated for monitoring DNA excision events [72,73]. The *codA* gene required a kill curve be determined for both 5-fluorocytosine (5FC) and 5-fluorouracil (5FU (Supplemental Figure S6). In the presence of a functional *codA* gene the nontoxic 5FC is converted to it toxic form 5FU. The toxin 5FU is a DNA chain-terminating compound that will stunt or kill germinating seed at very low concentrations.

**Figure 3:**
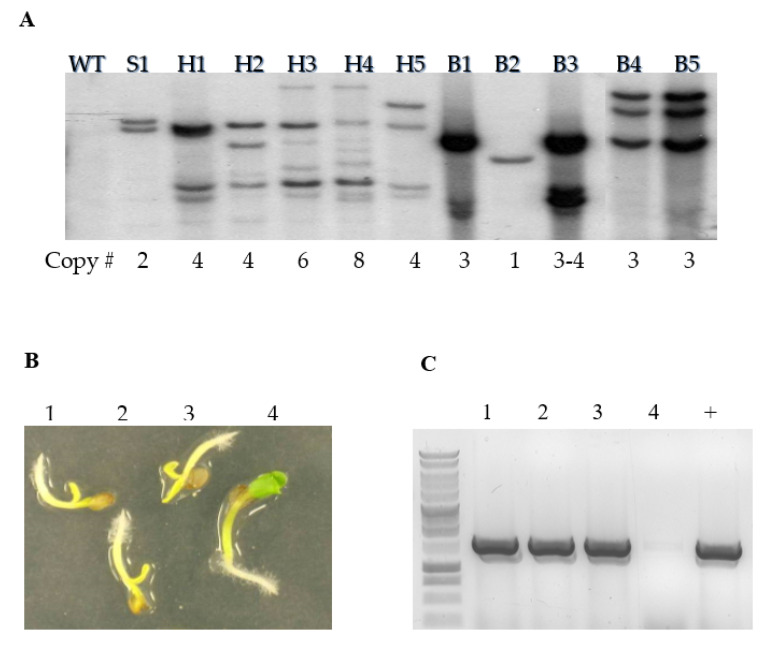
Biotechnology. (**A**) Southern blot analysis of camelina obtained from selection on sulfadiazine 150 mg/L (S1), hygromycin 30 mg/L (H1-5) and Basta 25 mg/L (B1-5). (**B**) Germination experiment of CTAG-35HC #2 T_1_ *Camelina* seeds on MS media containing 500 mg/L of 5-FC. Seedlings 1, 2, and 3 had a yellow, stunted phenotype, while seedling 4 was green and had healthy growth. (**C**) DNA was extracted from seedlings and analyzed using PCR with primers specific for the *codA* transgene.

Employing the Lu and Kang [41] method, a series of binary vectors were transformed into the camelina cultivar Suneson (Supplemental Figure S1). These vectors were designed with a positive/negative marker cassette flanked by recombinase recognition sites for use as RMCE founder lines [56] and capable of gene stacking. During the hygromycin (*hptII*) selection trial, 13 transformed lines were recovered. All lines contained a high T-DNA copy number (4 or more), as determined by Southern blot analysis (SBA) (Figure 3A). Plants under selection appeared sickly and were difficult to identify against background (wild-type) growth. However, plants did recover once transferred to soil. Glufosinate (bar) selection identified 14 plants that showed both low and multiple copy lines by SBA (Figure 3A). Identified plants appeared healthy under selection. Sulfadiazine revealed one line from initial trials; it was determined to be a 2 copy T-DNA insertion event by SBA and the plant appeared healthy (Figure 3A). The final positive selection marker tested was *nptII*. While a kill curve range was determined for the antibiotics kanamycin and G418 (Geneticin) (Supplemental Figure S2), use of the *nptII* selection gene, driven by the double enhanced 35S promoter (pCTAG-GCN), was only sufficient to produce a single resistance transgenic camelina from ~20,000 seeds screened.

To determine if this was a failure of the marker or poor rates of transformation, a second binary vector previously used for transformation, pCTAGV-KCN3 [72] was used. Seed selection was split onto either kanamycin selection or DsRed (visual) selection for germinating seedlings. The fluorescent DsRed selection marker under a constitutive promoter has previously been used to identify transformed seed based on fluorescence [41]. Five plants were obtained using visual DsRed selection, while zero plants were obtained from kanamycin selection of ~1000 plated seeds. 

For *codA* negative selection testing, T_2_ seeds from the aforementioned hygromycin, sulfadiazine and glufosinate positive selection studies were germinated in the presence of 500 mg L^−1^ 5FC. Results can be seen in Figure 3B, C and indicate that in the presence of a functional *codA* gene and the selective agent 5FC, camelina plants appear to grow yellow and stunted as compared to a null segregating sibling from the same transformation event that appears green and robust. 

In an attempt to determine whether any of the 41 lines investigated were viable for biotechnological manipulation we chose the line PI 311735 (due to its large seed and high oil content) for transformation using the pCTAG-GBC binary vector and glufosinate (bar) selection. Rates of transformation were similar to the control accession Suneson for production of transgenic plants through the floral dip method described [41]. While not overly effective we were able to obtain transgenic camelina plants with an efficiency of 0.6% using bar selection. PCR was used to provide molecular verification of transgenic camelina obtained from the PI 311735 and Suneson lines (Supplemental Figure S7). 

## 3. Discussion

The genus *Camelina* is composed of 11 species [74] but as of 2011, when seeds for this study were obtained, only five species: (*C. sativa*, *C. microcarpa*, *C. rumelica*, *C. alyssum*, and *C. hispida*) were present in the germplasm of the IPK (Institute of Plant Genetics and Crop Plant Research, Gatersleben, Germany) and the USDA-NGRP repositories. Among them, only *C. sativa* and *C. microcarpa* are cultivated. Within *C. sativa*, three different subspecies, ssp. *pilosa*, ssp. *sativa* and ssp. *foetida*, have been described [75]. 

Chromosome analysis of the 41 accessions (Table 6) displayed an interesting set of results. First, a single accession PI 650152 was found to contain an *n* = 13 and may have been mis-classified, although it appears to display phenotypic characteristics similar to *Camelina sativa* (L.) Crntz. Next, a split in chromosome number was obtained and it appears that 75.6% (31/41) lines have the predicted *n* = 20 chromosome number while 24.3% (10/41) have an *n* = 19. Previous results indicate that *Camelina sativa* (L.) Crntz. is an allohexaploid plant and in 2006, the Snowdon lab [50] demonstrated through the use of 157 AFLP marker linkage map and 3 Brassica SSR markers that the chromosome number of camelina was *n* = 20. These results were confirmed when the genome was sequenced, and a genome size of 750 Mbp was reported [49,76] for two different cultivars—accessions used were unpublished. It appears that triplication of the camelina genome occurred through whole genome duplication by either autopolyploidization or allopolyploidization. Though an autopolyploidy event triplicating a single diploid genome would result in an autohexaploid with a haploid genome of *n* = 18, 21, or 24 chromosomes depending on a starting genome chromosome count of *n* = 6, 7, or 8, respectively. However, camelina has reported chromosome counts of *n* = 6, 7, 10, 13, 18, 19, 20 [76,77]. 

Based on previous reports, *n* = 20 appeared to be the most common value and agrees with the recent AFLP marker linkage map and sequencing data. However, an *n* = 20 chromosome count would be difficult (although not impossible) to achieve through a single event triplicating a diploid genome. Triplication of the camelina genome from two allopolyploidy events, resulting in first a tetraploid followed by a second polyploid mating to produce a hexaploid, similar to the origin of cultivated 6-row wheat, is more likely. From previous research it appears that hybridization via outcrossing to related species is possible within the Brassicaceae family [78,79]. Taking into consideration the reported chromosome counts of various camelina related species, an initial allopolyploidy hybrid cross resulting from two diploid parental species where one was *n* = 6 and the other *n* = 7 could contribute to the production of a tetraploid genome with 13 chromosomes and would explain cultivar PI 650152. This is possible considering that related species *C. laxa* and *Camelina spp*. have *n* = 6, *C. hispida* has *n* = 7, and *C. rumelica* has *n* = 13 chromosome counts [80]. Following this logic, a second allopolyploidy hybridization event producing hexaploid progeny could be achieved by mating the tetraploid progeny (*n* = 13) with either of the two starting parental lines where a 13 + 6 cross would result in an *n* = 19 and a 13 + 7 event would result an *n* = 20 chromosome count. These hypothetical crosses would explain the varied chromosome counts documented in numerous camelina publications. The hypothetical crossing scenario is further supported by a recent publication [81] where the genetic diversity of the camelina genus was assessed across 54 accessions representing five species through RADseq, ITS sequencing, and flow cytometry. Results of the investigation infer that an (*n* = 6 + 7 + 7) hybridization is possible. The allopolyploid hypothesis is also supported by the observation that *C. sativa* demonstrates diploid inheritance [41,48], as would be expected for an allopolyploid [82]. A hexaploid *C. sativa* could also be derived from the combination of an autotetraploid and a diploid species if, in the autopolyploid genome, homologous chromosomes differentiated, so the subsequent chromosome-specific pairing mimicked an allopolyploid genome in its diploid inheritance patterns [82]. Regardless of its evolutionary path, the *C. sativa* genome appears organized in three redundant and differentiated copies and can be formally considered to be an allohexaploid. Our results support previous research and add to it that two hexaploid combinations exist within the known NGRP accessions *n* = 6 + 7 + 7 (20) and *n* = 6 + 7 + 6 (19).

From an agronomic point of view, *C. sativa* ssp. *sativa* and *Camelina sativa* ssp. *Pilosa*, sometimes termed ‘winter’ camelina, seem to be the most promising subspecies for abundant seed production, where 8 of the 9 cultivars examined had an above average seed count (4043 to 9225) when compared to the accessions as a whole (3328), see Table 7. These winter camelina subspecies are usually sown in autumn, since they require vernalization in order to attain stem elongation and subsequent flowering, while *Camelina sativa* ssp. *foetida* (aka., ‘spring’ Camelina) does not require vernalization and can be sown in both autumn and spring. All cultivars examine had an early emergence phenotype (2 days on average) that appears characteristic of the species in general, see Table 1. Bolting or time to flowering and time to dry are agronomic traits of importance, with the NGRP accessions ranging from 18 to 35 days and 52 to 72 days, respectively, see Table 3 and Table 4. From data observed rapid maturing and drying accessions appear to be PI 650145 and PI 650146. These lines may offer an opportunity to cultivate rapid cycling genotypes for double cropping utilization. 

Total seed weight for the various accessions ranged from a minimum of 1.45 g (PI 304270) to a maximum of 5.06 g (PI 311735). The oil content in dry weight seeds ranges between 23.6% (PI 650152) and 44.1% (PI650115) and consists of approximately 54% polyunsaturated, 34% monounsaturated, and 12% saturated fatty acids. The most abundant poly-unsaturated fatty acids are linoleic acid (C18:2), ranging from 10.5% to 16.4%, and linolenic acid (C18:3), ranging from 23.5% to 36.2%. The mono-unsaturated erucic acid (C22:1) is of importance for feed, with a maximum value of <2% allowed. Only one line, PI 650140, falls within this parameter at 1.83%, (Supplemental Table S1) and potentially provides useful breeding stock for improvement to camelina as an animal feed. Taken as a whole accession PI 311735 appears to provide an excellent set of agronomic traits for an oil seed crop. The cultivar has one of the earliest bolting times, at 18 days and a better than average drying time at 60.8 days. However, its most outstanding characteristics are its large total seed weight at 5.06 gram per plant and its above average oil content, measured at 38.2%. Accession PI 311735 was further tested for potential genetic manipulation through the agrobacterium ‘flora dip’ transformation technique in an attempt to produce a transgenic plant. Our lab successfully produced six transgenic plants as verified by seedling selection on glufosinate and confirmation by PCR. These results validate that this accession could undergo genetic modification.

From our examination the selectable marker bar (glufosinate) still appears to be the best selection system available for camelina genome modification. However, our research indicates that *sulI* (sulfadiazine) may be a viable option. Unfortunately, a more thorough study will be needed to validate this claim. The negative selectable maker gene *codA* was successful in inhibiting seedling growth in the presence of 5FC. Seedlings could even be rescued from the 5FC selection plate and grown to produce viable plants (data not shown). This will provide a useful selection tool for monitoring DNA excision events for techniques such and CRISRP and RMCE. 

## 4. Materials and Methods

Throughout the phenotypic descriptions, growth stages of camelina sativa presented are subtitled according to the two-digit BBCH scale [67] in the text. In the present investigation, plants were grown and evaluated under non-crowded greenhouse conditions in the California Bay area and therefore may not perfectly reflect more stressful field conditions in other parts of the country.

Table 9 presents accessions used listed according to the NGRP center designation, countries of origin and other names associated with these cultivars. 

### 4.1. Early Emergence Studies

After seeds were initially harvested, they were dried at 30 °C for one week and then weighed. To test to rate of germination, seed were imbibed on sterile 3 mm Whatman paper saturated with purified MQ water. It was observed that all viable seed for every cultivar formed a mucosal/gelatinous coat within the first 5 minutes of being imbibed. Seeds were placed on growth racks at 24 °C and observed for radical emergence. Vernalization was required for 9 of the 41 cultivars and these are indicated by asterisks at the end of their accession number (Table 1). These ‘winter’ types remained at the rosette stage indefinitely under greenhouse conditions, if not first cold-treated for the required vernalization time period to induce its bolting capacity. Therefore, all accessions were sown on moist soil and kept in the dark at 4 °C for 14 days. 

### 4.2. Greenhouse Phenotype Studies

Seeds were imbibed in water for 1 h prior to sowing in soil. For each accession, 5 seeds were sown per pot on the surface of damped soil treated with Gnatrol (3–12 pots were sowed depending on accession viability). All accessions once sown were kept in the dark at 4 °C for 14 days prior to placing in the greenhouse. Greenhouse growth conditions consisted of 18 h light, 6 h dark cycles at 26 °C and 24 °C, respectively. Whole plant measurements were taken every 3–4 days. Seeds were harvested when dry. Three to twelve pots were planted per accession depending on seed viability.

### 4.3. Seed Quality Traits

The weight of thousand seeds (TSW—thousand seed weight) was determined by measuring three replicates of thousand seeds each. Seeds were counted manually and weighed to the nearest 0.1 mg. Seeds were measured manually to the nearest 0.01 mm.

### 4.4. Seed Quality Trait Statistics

ANOVA and TukeyHSD statistical analyses of oilseed yield traits were performed in R (version 3.6.3) using aov() and TukeyHSD() functions. Prior to analyses, accessions with mixed traits (designated with A/B) and/or missing values were removed for a balanced design with *n* = 3 measurements per accession. Residual plots and Shapiro–Wilk normality tests were done to inspect ANOVA assumptions of normality and homogeneity of variance, and a square root transformation was implemented for total seed weight.

### 4.5. Oil Extraction and Weight to Volume Determination

Oil content for Camelina seeds (sample size 0.4–0.6 g, weighed to four decimal places) was determined non-destructively using a Bruker seed analyzer (Fremont, CA, USA) calibrated for Camelina, with each determination done in triplicate. From each Camelina accession, three samples (0.5 g) of dry seeds were ground with hexane (0.1% BHA) in a glass homogenizer (1.5 mL g^−1^ tissue) and poured into a 16 × 100 mm screw cap tube with a Teflon-lined screw cap. The solution was agitated for 30 min. The extract was then centrifuged with desktop Dynac centrifuge (Becton, Dickinson and Company) for 10 min at 1000 rpm (140 g) and the supernatant collected. The extraction procedure was repeated on the sediment. The hexane was evaporated under nitrogen and the residue dissolved in 1 mL of hexane (0.1% BHA) for further analysis.

### 4.6. Camelina Chromosome Squashes

For chromosome counts, 15 seed of each accession were germinated on moist filter paper in a 27°C growth chamber. Root tips were collected 5 days after germinated and were pretreated with 0.05% colchicine in 2% (*v/v*) DMSO for 4 h [78]. Root tips were then fixed overnight in 3:1 ethanol/glacial acetic acid. Slide preparations were made by digesting root tips for 30- to 60-min with 0.05 g L^−1^ Onuzuka R-10 cellulase and 0.01 g L^−1^ pectolyase Y-23 (Phytotechnology Labs) in 0.01 M citrate buffer pH 4.8 prior to. Digestion time varied according to the thickness and degree of lignification of the roots. Squashes were prepared according to Kirov et al., [83] and counts and were mounted in VECTASHIELD (Vector Laboratories) antifade mounting medium with DAPI (4,6-diamidino-2-phenylindole). Slide preparations were visualized under an Olympus BX51 fluorescent microscope. 

### 4.7. COT Value Determination 

The COT analysis is a technique that provides a way to measure DNA reassociation kinetics and gives a measure of repetitive DNA content per genome [54]. The procedure used to analyze nuclear DNA content in plant cells was modified from [84]. Briefly, the procedure consists of preparing suspensions of intact nuclei by chopping of 50 mg plant tissues in MgSO_4_ buffer mixed with DNA standards and stained with propidium iodide (PI) in a solution containing DNAase-free-RNAase. Fluorescence intensities of the stained nuclei are measured by a flow cytometer. Values for nuclear DNA content are estimated by comparing fluorescence intensities of the nuclei of the test population with those of an appropriate internal DNA standard that is included with the tissue being tested. Nuclei from *Arabidopsis thaliana* (0.36 pg/2C) was used as the internal standard. The pellet is suspended by vortexing vigorously in 0.5 mL solution containing 10 mM MgSO_4_.7H_2_O, 50mM KCl, 5 mM Hepes, pH 8.0, 3 mM dithiothreitol, 0.1 mg/mL propidium iodide, 1.5 mg/mL DNAse free RNAse (Rhoche, Indionapolis, IN, USA), and 0.25% Triton X-100. The suspended nuclei are withdrawn using a pipettor, filtered through 30-µm nylon mesh, and incubated at 37 °C for 30 min before flow cytometric analysis. Suspensions of sample nuclei is spiked with suspension of standard nuclei (prepared in above solution) and analyzed with a FACScalibur flow cytometer (Becton-Dickinson, San Jose, CA, USA). For each measurement, the propidium iodide fluorescence area signals (FL2-A) from 1000 nuclei are collected and analyzed by CellQuest software (Becton-Dickinson, San Jose, CA, USA) on a Macintosh computer. The mean position of the G0/G1 nuclei peak of the sample and the internal standard are determined by CellQuest software. The mean nuclear DNA content of each plant sample, measured in picograms, are based on 1000 scanned nuclei.

### 4.8. Plant Transformation

Modified from Lu and Kang [41]. From a single colony of *Agrobacterium*, grow 3 mL starter culture overnight at 28 °C. Inoculate 300 mL large-scale culture with starter culture and grow overnight at 28 °C with agitation. Collect cells by centrifugation, then suspend cells in transformation media (0.5X MS salts, 1X Gamborg vitamins, 50 g/L Sucrose, 0.01 mg/L BAP, 20 mg/L acetosyringone, 0.5 mL/L Silwet 77). Submerge the initial Camelina inflorescences into *Agrobacterium* suspension, and then swirl flowers gently in solution and vacuum infiltrated for 3 min. Wrap flowers in plastic wrap and store overnight in darkened room. Next day, unwrap flowers, and return plants to greenhouse to mature. Collect seed, then select transformants as described.

### 4.9. Plant Selection

Antibiotics, including kanamycin, G418 (*nptII*), hygromycin (*hptII*), and sulfadiazine (*sulI*), and the herbicide glufosinate (bar), to determine an effective concentration for routine for a floral dip protocol and positive seed selection. In addition, 5-fluorocytocine (5FC) and 5-fluorouracil (5FU) were investigated for negative selection. It was determined that a concentration of 200 mg L^−1^ for kanamycin, 30 mg L^−1^ for G418, 30 mg L^−1^ for hygromycin, 150 mg L^−1^ for sulfadiazine and 15 mg L^−1^ for glufosinate ammonium were effective at inhibiting growth of seedlings (Supplemental Figures S1–S5). Camelina was tolerant of 5FC up to 1000 mg L^−1^ but sensitive for 5FU at concentrations as low as 20 mg L^−1^ (Supplemental Figure S6). 

## 5. Conclusions

From our phenotypic evaluation of the 41 camelina accessions obtained from National Genetic Resources Program (NGRP) center, we identified a number of lines with potentially useful traits. For example, accession PI 311735, while providing only an average number of seed per plant (4953) produced the greatest yields in overall seed weight at 5.06 g per plant. This accession also had one of the highest mean oil contents per TSW at 38.2% and was seen to be faster than average for days to maturity and drying. As results were so encouraging with this line, its use for biotech application was also explored. It was observed that this accession was capable of transformation via traditional floral-dip technology and that glufosinate was an effective agent for seed selection. Thus, observation indicates that accession PI 311735 is a potential line for both breeding and biotech use. Another line of interest was observed through biochemical analysis of the oil, where it was discovered that accession PI 650141 had an erucic acid concentration of 1.83%, which is below the 2% as required for food consumption. Making this another potentially useful line for breeding efforts. Of interest was the split seen in the camelina population for chromosome number between *n* = 19 (24.3%) and *n* = 20 (75.6%). Our group hypothesized that camelina in its current hexaploidy form may have originated from two divergent but related pathways. In short both events could have begun with an *n* = 6 (*C. laxa)* and *n* = 7 (*C. hispida)* hybridization producing *n* = 13 (*C. rumelica*) like species. Then diverged with the second hybridization of *n* = 13 to one or the other original parent such that *n* = 6 + 7 + 6 (19) or 6 + 7 + 7 (20). From the biotechnological studies, it was discovered that the *codA* gene worked very efficiently at stunting camelina growth in the presences of 5FC. This should provide a valuable resource for techniques, such as RMCE or CRISPR, where the removal of DNA is a required component for both strategies. Finally, even though the *sulI* selection marker gene only produced a single plant that plant has been shown resistant to sulfa based herbicides (data not shown) and may provide farmers with a way to control weeds while cultivating camelina using conventional treatments. Data is presented in an uncompressed format in both tables for manuscript discussion and as raw data in XLS spreadsheet (Supplemental Tables S1–S4) format. With the variability seen within this collection it is our hope that this information will help direct breeding or biotechnological programs for camelina’s future use as biofuel and/or meal sustainable crop.

## Figures and Tables

**Table 1:** Early emergence.

Accessions ^**^	Vernalize ^a^	Radical Emerge (days) ^b^	Shoot Emerge (days) ^c^	Root Length (mm) ^d^	STDev	Hypocotyl Length (mm) ^e^	STDev	Cotyledon Emerge ^f^
PI 258366	N	1	2	22.0	4.9	6.4	0.5	60%
PI 258367	N	1	2	16.0	2.9	5.8	0.8	44%
PI 304269	N	1	2.2	27.8	7.5	7.8	2.3	25%
PI 304270	N	1	2	28.0	4.0	8.2	0.8	75%
PI 304271	N	1	2	20.0	4.3	7.4	0.5	100%
PI 311735	N	1	2	30.2	5.6	7.4	1.5	49%
PI 311736 *	Y	1	2	22.4	8.1	7.4	0.5	91%
PI 597833A	N	1	2	19.6	3.8	4.6	0.9	55%
PI 597833B	N	1	2	23.0	6.6	5.4	1.1	59%
PI 633192	N	1	1.8	27.2	3.1	6.4	0.5	96%
PI 633193*	Y	1	2	22.4	3.3	7.8	0.8	0%
PI 633194	N	1	2	26.2	2.8	10.0	0.7	81%
PI 650140	N	1	2	34.6	5.6	8.0	1.0	96%
PI 650141	N	1	2	32.6	3.8	7.8	1.5	60%
PI 650142A	N	1	2	17.2	3.5	8.8	0.8	6%
PI 650142B	N	1	2	25.6	2.9	7.2	1.1	88%
PI 650143 *	Y	1	2	5.4	1.5	2.8	0.8	3%
PI 650144	N	1	2.2	24.6	11.1	7.4	1.5	70%
PI 650145	N	1	2	30.4	9.6	8.2	0.8	29%
PI 650146	N	1	2	23.0	6.7	8.4	1.1	61%
PI 650147	N	1	2	20.2	4.8	9.0	0.7	24%
PI 650148	N	1	2	23.0	3.3	7.6	0.5	100%
PI 650149	N	1	2	26.8	6.6	8.2	0.8	77%
PI 650150	N	1	2	38.2	4.3	8.6	0.5	85%
PI 650151	N	1	2	33.4	3.5	9.0	1.0	75%
PI 650152 *	Y	1	2	8.8	1.3	2.2	0.4	22%
PI 650153	N	1	2	38.0	7.2	8.8	1.3	0%
PI 650154	N	1	2	26.4	2.8	8.0	0.7	96%
PI 650155 *	Y	1	2	17.6	3.5	7.0	1.0	98%
PI 650156	N	1	2	31.2	6.3	9.4	0.5	100%
PI 650157A *	Y	1	2	22.6	2.7	5.4	0.5	69%
PI 650157B	N	1	2	24.2	1.9	5.4	1.1	14%
PI 650158A *	Y	1	2	40.4	5.1	7.4	0.5	88%
PI 650158B	N	1	2	26.2	6.3	8.2	0.8	90%
PI 650159	N	1	2	37.4	15.1	7.8	1.3	80%
PI 650163	N	1.2	1.8	22.0	2.7	7.2	1.3	24%
PI 650164	N	1	2	30.8	7.0	8.6	1.1	100%
PI 650165	N	1	2	36.8	13.8	8.8	1.8	94%
PI 650166	N	1	2	28.4	5.9	8.0	1.2	7%
PI 650167 *	Y	2	3	13.8	1.5	3.8	0.8	66%
PI 650168 *	Y	1	2	20.6	3.6	5.6	0.5	89%
Suneson	N	1	2	29.8	10.8	10.8	0.8	75%

* Indicates accessions that required vernalization in order to flower. Also described as ‘winter’ accessions. ** A sample size (*n*) of 3–12 was used per cultivar. A and B designation were given to accession obtained from the NGRP center that contained more than two unique phenotypes. ^a^ Defined as requirement to keep seed for 8 weeks at 4 °C before being able bolt or flower. ^b^ Defined as time it takes for root tip to after sowing seeds on wet filter paper. ^c^ Defined as time it takes for shoot to emerge from the seed coat. ^d^ Root length measured 4 days after sowing seeds on wet filter paper. ^e^ Hypocotyl length measured 4 days after sowing seeds on wet filter paper. ^f^ Defined as the percentage of plant that have unfurled cotyledons greater than 50% within 4 days after sowing.

**Table 2:** Branch and leaf morphology.

Accession ^**^	Leaf Shape	Leaf Margin ^a^	Leaf Points ^b^	Total Points	Branch Pattern ^c^
PI 258366	subulate	serrate	4 + 4	8	W
PI 258367	lanceolate	smooth	NA	NA	W
PI 304269	lanceolate	smooth	NA	NA	W
PI 304270	lanceolate	spiny	7 + 7	14	W
PI 304271	lanceolate	spiny	5 + 5	10	W
PI 311735	lanceolate	spiny	6 + 5	11	W
PI 311736 *	lanceolate	mildly serrate	6 + 6	12	Y
PI 597833A	linear	serrated	9 + 9	18	W
PI 597833B	lanceolate	spiny	6 + 5	11	W
PI 633192	lanceolate	spiny	6 + 6	12	W
PI 633193 *	lanceolate	smooth	NA	NA	X
PI 633194	lanceolate	spiny	7 + 8	15	W
PI 650140	lanceolate	spiny	5 + 5	10	X
PI 650141	lanceolate	spiny	6 + 5	11	W
PI 650142A	linear	serrate	7 + 6	13	W
PI 650142B	lanceolate	spiny	6 + 6	12	W
PI 650143 *	subulate	mildly serrate	6 + 6	12	X
PI 650144	lanceolate	mildly serrate	8 + 8	16	W
PI 650145	lanceolate	smooth	NA	NA	W
PI 650146	lanceolate	smooth	NA	NA	W
PI 650147	lanceolate	spiny	6 + 7	13	W
PI 650148	lanceolate	smooth	NA	NA	W
PI 650149	lanceolate	spiny	8 + 7	15	W
PI 650150	lanceolate	smooth	NA	NA	W
PI 650151	lanceolate	smooth	NA	NA	W
PI 650152 *	lanceolate	serrate	7 + 7	14	X
PI 650153	lanceolate	spiny	5 + 5	10	W
PI 650154	lanceolate	spiny	5 + 5	10	W
PI 650155 *	subulate	mildly serrate	7 + 7	14	W
PI 650156	lanceolate	serrate	9 + 9	18	W
PI 650157A *	subulate	mildly serrate	8 + 8	16	X
PI 650157B	subulate	smooth	NA	NA	Z
PI 650158A *	lanceolate	spiny	7 + 7	14	W
PI 650158B	lanceolate	smooth	NA	NA	W
PI 650159	lanceolate	mildly serrate	6 + 5	11	X
PI 650163	subulate	spiny	13 + 21	34	W
PI 650164	lanceolate	serrate	6 + 7	13	Y
PI 650165	subulate	spiny	3 + 4	7	W
PI 650166	lanceolate	spiny	9 + 5	14	W
PI 650167 *	lanceolate	smooth	NA	NA	W
PI 650168 *	lanceolate	mildly serrate	6 + 6	12	Z
Suneson	lanceolate	spiny	8 + 6	14	W

* Indicates accessions that required vernalization in order to flower. Also described as ‘winter’ accessions. ** A sample size (*n*) of 3–12 was used per cultivar. See Supplemental Table S2 for specific values. A and B designation were given to accession obtained from the NGRP center that contained more than two unique phenotypes. ^a^ Spiny margin, defined as having a series of sharp stiff points; serrated margin, defined as having a series of wave like forward pointed teeth around the entire leaf edge; smooth margin, defined as no projections around the outside of the leaf. ^b^ Points are defined as short needle like projections or spines from the edge of the leaf. ‘4 + 4’ defined as points on ‘right’ side of leaf + points on ‘left’ side of leaf. ^c^ W—Top heavy branching often seen with secondary branching; X—Branched length of main stem with few secondary branches; Y—Tiller where all branches originate from the base and contain few secondary branching; Z—Chaos where branches are seen originating everywhere and a dominant stem was not observed.

**Table 3:** Flowering development.

Accession ^**^	Bolting (days) ^a^	STDev	Flowering (days) ^b^	STDev	Height (cm) ^c^	STDev	Width (cm) ^d^	STDev	1º Floral Meristem ^e^	STDev
PI 258366	21.0	0.0	29.8	1.1	48.3	3.3	22.6	1.9	18.4	3.3
PI 258367	20.3	1.4	34.3	4.5	49.4	5.1	18.9	4.0	12.9	2.7
PI 304269	23.0	0.0	37.4	1.3	50.9	6.1	21.6	1.5	13.2	1.6
PI 304270	23.0	0.0	37.4	1.3	50.9	6.0	22.6	1.5	13.2	2.2
PI 304271	21.0	0.0	29.0	0.0	49.6	3.8	23.0	2.0	13.4	1.1
PI 311735	18.1	1.2	32.6	3.5	50.7	8.8	18.1	4.9	10.1	4.2
PI 311736 *	24.0	0.0	30.6	4.3	30.8	14.0	25.1	0.5	11.0	2.9
PI 597833A	21.0	0.0	32.3	4.9	38.8	7.5	20.6	2.8	10.3	1.2
PI 597833B	20.0	2.1	33.0	3.0	46.8	8.5	18.2	3.7	9.6	2.2
PI 633192	23.0	4.0	31.3	4.5	36.4	4.5	24.3	2.1	13.3	2.9
PI 633193 *	19.2	2.7	33.2	4.9	43.5	10.3	17.4	5.9	14.4	5.7
PI 633194	19.9	1.6	33.9	4.2	50.0	3.5	17.8	2.6	10.1	4.2
PI 650140	20.6	1.1	32.4	3.2	49.5	7.1	20.9	2.3	11.9	2.1
PI 650141	21.0	0.0	29.0	0.0	41.0	5.1	18.8	0.8	11.0	1.6
PI 650142A	21.8	1.0	31.3	3.1	38.5	4.1	22.8	1.5	12.0	1.9
PI 650142B	22.0	1.3	33.3	3.7	45.6	8.8	21.6	1.6	12.4	1.7
PI 650143 *	35.0	0.0	38.0	0.0	32.5	0.7	20.0	2.8	9.0	0.0
PI 650144	24.3	3.2	38.0	2.8	47.4	6.9	20.8	4.8	10.8	1.4
PI 650145	23.0	0.0	35.6	1.3	44.2	4.4	20.8	1.1	10.6	0.5
PI 650146	23.0	0.0	38.6	2.5	49.0	6.0	23.0	3.7	10.2	1.9
PI 650147	23.0	0.0	38.6	1.3	48.6	1.5	24.4	1.7	12.4	1.1
PI 650148	23.0	0.0	41.2	4.1	47.0	4.6	18.8	3.4	12.2	1.9
PI 650149	27.0	0.0	42.8	1.6	50.3	8.2	23.0	0.7	14.8	1.6
PI 650150	23.0	0.0	36.2	1.6	40.2	6.2	22.6	4.0	9.2	0.8
PI 650151	23.0	0.0	36.8	1.6	56.5	10.8	25.6	1.5	11.0	1.0
PI 650152 *	30.0	3.1	37.4	1.3	46.8	8.9	25.0	5.9	12.6	1.8
PI 650153	20.0	0.0	33.6	3.3	51.6	16.1	22.8	0.8	13.2	4.1
PI 650154	20.0	0.0	36.0	0.0	60.6	10.9	19.8	1.5	14.2	4.5
PI 650155 *	21.0	0.0	29.2	1.6	39.2	7.0	26.6	2.3	10.4	1.3
PI 650156	20.6	0.5	35.4	0.5	54.4	8.0	18.4	4.3	10.3	2.2
PI 650157A *	21.6	1.3	28.6	1.3	28.6	7.1	21.6	2.7	11.0	3.1
PI 650157B	24.0	1.9	33.5	2.8	40.7	9.7	15.8	1.6	10.0	1.5
PI 650158A *	21.0	0.0	31.0	0.0	36.6	5.6	25.2	3.8	10.6	3.3
PI 650158B	22.2	2.7	31.2	3.9	47.2	3.4	22.8	2.9	9.4	0.9
PI 650159	20.0	0.0	37.0	2.2	62.1	6.4	23.8	1.9	11.2	1.6
PI 650163	22.4	4.4	29.8	1.1	44.3	14.9	24.0	1.0	11.6	2.9
PI 650164	21.0	0.0	29.0	0.0	39.7	5.5	22.2	2.2	11.4	1.1
PI 650165	21.0	0.0	29.0	0.0	58.4	4.3	20.8	1.3	16.0	2.1
PI 650166	21.0	0.0	33.2	3.8	43.1	7.8	17.7	2.7	12.4	3.9
PI 650167 *	33.8	3.4	39.0	2.0	23.0	8.8	22.3	2.6	9.8	1.5
PI 650168 *	23.0	1.7	29.0	1.7	29.7	6.5	24.3	0.6	11.7	2.1
Suneson	22.1	1.2	36.8	4.1	50.5	4.2	20.8	5.0	12.0	3.5

* Indicates accessions that required vernalization in order to flower. Also described as ‘winter’ accessions. ** A sample size (*n*) of 3–12 was used per cultivar. See Supplemental Table S2 for specific values. A and B designation were given to accession obtained from the NGRP center that contained more than two unique phenotypes. ^a^ Defined as the rapid development of the first flower stalk or central stem. ^b^ Defined as time when greater than 50% of all floret within the initial composite flower are open. ^c^ Maximum height of plant as determined at initial flowering. ^d^ Maximum width of plant as determined at initial flowering. ^e^ Primary inflorescence is composed of a number of florets (one of the small flowers) making up a composite flower head.

**Table 4:** Plant development.

Accession ^**^	Maturity (days) ^a^	STDev	Height (cm) ^b^	STDev	Width (cm) ^c^	STDev	Dry (days) ^d^	STDev
PI 258366	43.2	1.6	80.4	4.2	39.8	7.2	53.6	2.2
PI 258367	48.3	5.2	79.1	5.5	41.8	6.4	54.7	2.3
PI 304269	49.8	1.6	82.5	1.4	32.8	3.4	62.0	0.0
PI 304270	50.4	1.3	83.4	3.4	38.6	4.3	62.0	0.0
PI 304271	42.0	0.0	76.6	2.7	38.4	5.5	52.0	0.0
PI 311735	48.3	5.2	74.3	6.8	33.1	8.8	60.8	7.2
PI 311736 *	52.0	0.0	71.4	6.9	49.8	13.8	66.0	0.0
PI 597833A	50.3	4.1	67.2	5.5	44.2	4.4	63.7	5.7
PI 597833B	48.3	5.2	80.4	6.8	39.0	4.0	60.8	7.2
PI 633192	44.5	5.0	72.5	3.7	31.8	5.7	53.8	3.5
PI 633193 *	52.0	0.0	73.0	9.2	57.4	21.1	66.0	0.0
PI 633194	48.3	5.2	71.8	7.3	35.9	8.7	64.4	10.3
PI 650140	47.7	5.3	77.0	6.8	48.6	15.2	60.0	7.5
PI 650141	42.0	0.0	74.6	3.8	36.2	7.3	52.0	0.0
PI 650142A	44.3	3.1	64.9	3.2	25.0	2.8	58.4	9.6
PI 650142B	45.0	4.2	80.6	3.1	39.9	3.2	54.9	4.9
PI 650143 *	63.0	0.0	81.5	2.1	51.5	9.2	66.0	0.0
PI 650144	54.0	1.7	78.1	6.5	40.1	4.0	69.9	3.2
PI 650145	49.8	1.6	82.1	4.9	37.4	3.4	72.0	0.0
PI 650146	54.2	1.8	82.6	9.2	45.8	9.9	72.0	0.0
PI 650147	52.6	2.2	82.3	5.9	37.8	8.5	71.4	1.3
PI 650148	56.8	4.1	78.8	6.2	34.8	5.4	71.4	1.3
PI 650149	58.0	0.0	79.3	8.3	32.2	0.8	72.0	0.0
PI 650150	49.8	1.6	78.6	6.3	33.6	4.7	72.0	0.0
PI 650151	49.2	1.6	88.3	5.1	32.8	2.5	62.0	0.0
PI 650152 *	57.4	3.1	81.6	8.3	38.2	14.7	66.0	0.0
PI 650153	44.0	0.0	75.6	5.4	11.4	1.7	72.0	0.0
PI 650154	48.6	1.3	84.6	6.6	29.4	0.9	72.0	0.0
PI 650155 *	51.4	1.3	73.4	4.4	47.6	4.6	66.0	0.0
PI 650156	50.5	2.1	76.5	8.2	32.5	8.0	68.1	5.2
PI 650157A *	52.0	0.0	67.2	7.7	59.2	14.3	66.0	0.0
PI 650157B	49.8	5.0	75.5	5.0	30.7	10.3	61.5	6.0
PI 650158A *	52.0	0.0	66.2	13.8	33.4	15.5	66.0	0.0
PI 650158B	43.8	4.0	82.6	0.9	27.4	5.2	56.0	8.9
PI 650159	49.4	3.1	85.2	0.8	25.4	2.7	69.2	3.8
PI 650163	45.4	4.8	78.6	11.1	35.8	3.9	56.0	0.0
PI 650164	42.0	0.0	72.0	6.8	30.0	3.5	52.0	0.0
PI 650165	42.0	0.0	85.4	3.2	36.6	4.8	52.0	0.0
PI 650166	47.9	5.0	72.4	4.1	36.6	4.6	62.0	9.5
PI 650167 *	62.0	2.0	69.5	8.2	37.3	9.0	70.0	0.0
PI 650168 *	52.0	0.0	69.7	1.2	61.0	3.6	66.0	0.0
Suneson	50.9	4.5	81.5	8.5	31.9	6.1	65.8	7.2

* Indicates accessions that required vernalization in order to flower. Also described as ‘winter’ accessions. ** A sample size (*n*) of 3–12 was used per cultivar. See Supplemental Table S2 for specific values. A and B designation were given to accession obtained from the NGRP center that contained more than two unique phenotypes. ^a^ Defined as time when greater than 50% of all flowers are open and seed set has begun. ^b^ Maximum height of plant as determined at full flowering and seed set has begun. ^c^ Maximum width of plant as determined at full flowering and seed set has begun. ^d^ Defined as time from maturity till time when seed pods are visibly brown and brittle to the touch.

**Table 5:** Seed values.

Accession ^**^	Mean Seed Weight (mg) ^a^	STDev	Mean Total Weight (g) ^b^	STDev	Est. Total Seeds per Plant ^c^	Mean Width(mm)	STDev	Mean Length(mm)	STDev	L:W Ratio	STDev
PI 258366	0.7576	0.21	3.2091	0.19	4236	0.87	0.08	1.83	0.15	2.12	0.04
PI 258367	0.8624	0.11	3.1424	0.50	3644	0.99	0.03	1.97	0.03	2.00	0.03
PI 304269	0.6608	0.07	1.5185	0.13	2298	0.88	0.08	1.87	0.03	2.17	0.16
PI 304270	0.4446	0.08	1.4478	0.27	3256	0.65	0.09	1.63	0.11	2.57	0.22
PI 304271	0.6799	0.10	3.4842	0.11	5125	0.88	0.05	1.72	0.09	1.95	0.06
PI 311735	1.0219	0.13	5.0617	0.48	4953	1.06	0.05	1.95	0.06	1.85	0.11
PI 311736 *	0.7931	0.13	3.7375	0.91	4712	0.90	0.05	1.70	0.01	1.90	0.11
PI 597833A	0.7468	n/a	2.9545	n/a	3313	0.89	0.04	1.80	0.04	2.03	0.08
PI 597833B	0.6486	0.03	3.1004	0.52	4780	0.87	0.05	1.65	0.05	1.90	0.12
PI 633192	0.6151	0.05	3.5219	0.19	5725	0.87	0.01	1.75	0.06	2.02	0.07
PI 633193 *	0.7759	0.01	4.4079	0.47	5681	0.90	0.03	1.76	0.03	1.95	0.03
PI 633194	0.6932	0.08	2.6860	0.57	3875	0.94	0.01	1.97	0.14	2.11	0.15
PI 650140	0.9130	0.07	3.0416	0.53	3331	1.02	0.02	2.12	0.05	2.09	0.07
PI 650141	0.5651	0.07	2.5286	0.32	4475	0.83	0.03	1.68	0.03	2.05	0.06
PI 650142A	0.6981	0.05	2.1806	0.24	3124	0.83	0.04	1.80	0.06	2.19	0.12
PI 650142B	0.5487	0.02	2.4079	0.46	4388	0.76	0.00	1.73	0.03	2.30	0.03
PI 650143 *	0.1916	0.01	1.7671	0.37	9225	0.70	0.01	1.31	0.00	1.87	0.04
PI 650144	0.6113	0.01	2.0327	0.06	3325	0.75	0.02	1.70	0.05	2.29	0.00
PI 650145	0.6894	0.00	1.9309	0.22	2801	0.82	0.03	1.78	0.03	2.18	0.08
PI 650146	0.8062	0.06	1.8001	0.42	2233	0.81	0.05	2.04	0.06	2.55	0.18
PI 650147	0.7902	0.05	1.7100	0.09	2164	0.80	0.02	2.03	0.02	2.56	0.11
PI 650148	0.6605	0.10	1.5805	0.13	2393	0.79	0.02	1.86	0.21	2.39	0.25
PI 650149	0.5511	0.06	1.5180	0.18	2754	0.73	0.06	1.64	0.04	2.26	0.14
PI 650150	0.9297	0.13	2.2508	0.13	2421	0.89	0.04	2.03	0.07	2.30	0.18
PI 650151	0.7199	0.04	1.6820	0.30	2336	0.77	0.01	2.01	0.06	2.62	0.11
PI 650152 *	0.3366	0.03	2.0645	0.27	6133	0.79	0.01	1.38	0.02	1.74	0.05
PI 650153	1.0473	0.10	1.6804	0.04	1604	0.93	0.03	2.01	0.02	2.17	0.08
PI 650154	0.8587	0.04	2.2814	0.18	2657	0.97	0.12	1.79	0.02	1.90	0.16
PI 650155 *	0.9102	0.03	4.2544	0.99	4674	0.94	0.02	1.70	0.05	1.82	0.03
PI 650156	0.6941	0.09	1.8880	0.09	2720	0.81	0.04	1.78	0.09	2.21	0.11
PI 650157A *	0.8465	0.09	3.4222	1.70	4043	0.91	0.06	1.69	0.06	1.89	0.06
PI 650157B	0.9095	0.07	2.3057	0.36	2535	1.00	0.09	1.97	0.06	1.99	0.21
PI 650158A *	0.9518	0.04	1.7637	0.55	1853	0.96	0.06	1.93	0.03	2.04	0.12
PI 650158B	0.7851	0.08	2.0477	0.34	2608	0.94	0.07	1.86	0.07	1.98	0.17
PI 650159	1.0456	0.05	2.6669	0.42	2551	1.03	0.02	1.99	0.07	1.94	0.06
PI 650163	0.8302	0.02	3.1996	0.20	3854	0.96	0.02	1.83	0.01	1.91	0.03
PI 650164	0.5916	0.14	1.9030	0.31	3217	0.89	0.09	1.77	0.08	2.02	0.16
PI 650165	0.7562	0.05	2.4967	0.26	3302	0.95	0.01	1.99	0.08	2.11	0.09
PI 650166	0.6815	0.11	2.9354	1.06	4307	0.90	0.04	1.83	0.05	2.05	0.06
PI 650167 *	0.2006	0.03	1.7985	0.75	8967	0.69	0.02	1.20	0.02	1.76	0.05
PI 650168 *	0.8577	0.13	4.9498	1.17	5771	0.96	0.02	1.73	0.06	1.81	0.04
Suneson	0.7845	0.13	2.9444	0.65	3753	0.98	0.09	1.94	0.06	2.00	0.15

* Indicates accessions that required vernalization in order to flower. Also described as ‘winter’ accessions. ** A sample size (*n*) of 10 was used per cultivar. See Supplemental Table S2 for specific values. A and B designation were given to accession obtained from the NGRP center that contained more than two unique phenotypes. ^a^ Defined as the average value of 1000 seed total weight divided by 1000 from 10 plants. ^b^ Defined as the average total weight of seeds from 10 plants. ^c^ Defined as mean total weight divided by the mean seed weight. See Supplemental Tables S1–S4 for specific values.

**Table 6:** Oil values.

Accession **	Mean OC (%) ^a^	16:0 Palmitic (%)	18:1n-9 Oleic (%)	18:2n-6 Linoleic LA (%)	18:3n-3 ALA (%)	20:1n-9 Gondoic (%)	TSW (g) ^b^
PI 258366	36.1	6.4	15.0	25.4	26.6	12.8	0.7576
PI 258367	35.5	7.6	14.6	24.8	27.9	12.6	0.8624
PI 304269	26.4	7.5	12.2	28.6	23.5	11.6	0.6608
PI 304270	26.4	6.7	14.1	23.6	28.7	13.4	0.4446
PI 304271	35.6	7.0	13.8	21.2	29.4	12.9	0.6799
PI 311735	38.2	7.0	12.2	25.8	26.2	12.9	1.0219
PI 311736 *	38.3	7.9	10.7	24.8	29.5	12.0	0.7931
PI 597833A	37.5	7.3	14.4	19.7	32.2	13.0	0.7468
PI 597833B	36.9	7.4	13.9	19.6	33.5	12.5	0.6486
PI 633192	34.7	7.1	13.7	23.1	26.7	14.4	0.6151
PI 633193 *	33.4	6.4	14.9	18.0	33.7	13.6	0.7759
PI 633194	29.6	8.3	12.2	27.4	30.1	10.5	0.6932
PI 650140	30.6	7.3	14.5	24.0	27.5	12.2	0.9130
PI 650141	33.4	9.5	15.7	28.4	30.4	10.7	0.5651
PI 650142A	32.7	8.6	13.8	23.7	29.7	10.9	0.6981
PI 650142B	31.5	7.4	13.0	24.8	30.9	11.9	0.5487
PI 650143 *	23.9	7.5	9.8	19.7	29.2	11.4	0.1916
PI 650144	30.5	7.6	14.2	23.7	27.9	13.3	0.6113
PI 650145	34.6	7.0	13.4	18.7	36.2	12.0	0.6894
PI 650146	29.3	7.4	14.1	25.6	27.8	10.7	0.8062
PI 650147	30.1	7.1	11.9	20.8	32.6	13.6	0.7902
PI 650148	26.9	8.7	10.1	21.9	33.2	12.0	0.6605
PI 650149	29.2	7.5	9.1	21.3	32.0	12.8	0.5511
PI 650150	31.9	6.5	10.3	20.2	35.2	12.6	0.9297
PI 650151	29.8	6.7	14.4	21.2	32.8	12.0	0.7199
PI 650152 *	23.6	8.7	17.1	17.3	24.9	16.4	0.3366
PI 650153	37.4	6.8	9.8	21.7	29.9	14.7	1.0473
PI 650154	33.9	6.2	10.1	19.8	33.9	13.2	0.8587
PI 650155 *	44.1	5.5	14.1	18.4	34.6	13.5	0.9102
PI 650156	35.9	6.4	11.0	19.1	34.3	13.5	0.6941
PI 650157A *	37.7	5.9	13.9	17.9	32.4	15.1	0.8465
PI 650157B	36.4	7.0	12.6	21.2	29.6	13.3	0.9095
PI 650158A *	35.7	7.2	13.8	16.1	32.8	14.3	0.9518
PI 650158B	34.8	7.1	11.4	21.6	29.2	14.5	0.7851
PI 650159	37.5	6.6	12.0	21.3	30.1	13.2	1.0456
PI 650163	39.6	6.3	13.6	20.8	32.5	13.1	0.8302
PI 650164	33.6	6.9	13.3	21.6	29.0	14.3	0.5916
PI 650165	31.9	7.5	12.5	27.6	23.5	12.6	0.7562
PI 650166	44.0	7.8	12.3	27.3	24.9	11.4	0.6815
PI 650167 *	25.3	6.8	12.2	21.4	29.6	12.8	0.2006
PI 650168 *	41.0	6.2	12.3	19.1	34.3	14.0	0.8577
Suneson	30.7	7.5	10.6	27.0	26.0	13.0	0.7845

* Indicates accessions that required vernalization in order to flower. Also described as ‘winter’ accessions. ** A sample size (*n*) of 3 was used per cultivar and mean values presented. See Supplemental Tables S1–S4 for specific values. A and B designation were given to accession obtained from the NGRP center that contained more than two unique phenotypes. ^a^ OC—oil content ^b^ TSW—Thousand Seed Weight.

**Table 7:** Chromosome number and COT value.

Accession	Chromosome # ^a^	COT Value—DNA (pg/2C) ^b^	STDev
PI 258366	40	1.63	0.015
PI 258367	38	1.66	0.016
PI 304269	40	1.58	0.008
PI 304270	40	1.55	0.032
PI 304271	40	1.66	0.012
PI 311735	38	1.64	0.012
PI 311736 *	40	1.62	0.007
PI 597833A	40	1.41	0.017
PI 597833B	40	1.38	0.099
PI 633192	40	1.60	0.008
PI 633193 *	40	1.61	0.016
PI 633194	40	1.61	0.029
PI 650140	40	1.62	0.022
PI 650141	38	1.60	0.020
PI 650142A	40	1.44	0.052
PI 650142B	40	0.58	0.017
PI 650143 *	38	1.29	0.097
PI 650144	38	1.25	0.094
PI 650145	40	1.33	0.083
PI 650146	38	1.33	0.070
PI 650147	40	1.37	0.027
PI 650148	40	1.27	0.034
PI 650149	40	1.30	0.040
PI 650150	40	0.98	0.072
PI 650151	40	1.43	0.031
PI 650152 *	26	1.15	0.095
PI 650153	40	1.20	0.063
PI 650154	40	0.97	0.088
PI 650155 *	40	1.42	0.083
PI 650156	40	0.83	0.093
PI 650157A *	40	1.35	0.014
PI 650157B	38	1.23	0.045
PI 650158A *	40	1.35	0.025
PI 650158B	40	0.97	0.043
PI 650159	40	1.46	0.015
PI 650163	40	1.66	0.027
PI 650164	38	1.62	0.020
PI 650165	40	1.58	0.018
PI 650166	40	1.67	0.023
PI 650167 *	38	1.61	0.036
PI 650168 *	38	1.48	0.015
Suneson	40	1.14	0.063

* Indicates accessions that required vernalization in order to flower. Also described as ‘winter’ accessions. A and B designation were given to accession obtained from the NGRP center that contained more than two unique phenotypes. ^a^ Date present as a 2*n* value. Sampling size ranged from 3–33 to determine chromosome counts. See Supplemental Table S2 for details. ^b^ COT value is defined as DNA reassociation kinetics and gives a measure of repetitive DNA content per genome. Sampling size was 1000 nuclei.

**Table 8:** A/B cultivar analysis.

Accession ^**^	COT (pg/2C) ^a^	Chrom # ^b^	Vernalize ^c^	Branch Pattern ^d^	Leaf Shape	Leaf Margin	Flowering (Days) ^e^	Height at Flowering (cm) ^f^	Florets in Initial Flower ^g^	Maturity (days) ^h^	Height at Maturity (cm) ^i^
PI 597833A	1.41	40	N	W	linear	serrate	33.0	38.8	10.3	50.3	67.2
PI 597833B	1.38	40	N	W	lanceolate	spiny	31.3	46.8	9.6	48.3	80.4
PI 650142A	1.44	40	N	W	linear	serrate	31.3	38.5	12.0	44.3	64.9
PI 650142B	0.58	40	N	W	lanceolate	spiny	33.3	45.6	12.4	45.0	80.6
PI 650157A *	1.35	40	Y	X	subulate	serrate	28.6	28.6	11.0	52.0	67.2
PI 650157B	1.23	38	N	Z	subulate	smooth	33.5	40.7	10.0	49.8	75.5
PI 650158A *	1.35	40	Y	W	lanceolate	spiny	31.0	36.6	10.6	52.0	66.2
PI 650158B	0.97	40	N	W	lanceolate	smooth	31.2	47.2	9.4	43.8	82.6

* Indicates accessions that required vernalization in order to flower. Also described as ‘winter’ accessions. ** A sample size (*n*) of 3–12 was used per cultivar. See Supplemental Table S2 for specific values. A and B designation were given to accession obtained from the NGRP center that contained more than two unique phenotypes. ^a^ COT value is defined as DNA reassociation kinetics and gives a measure of repetitive DNA content per genome ^b^ Sampling size ranged from 3–33 to determine chromosome counts. See Supplemental Table S2 for details. ^c^ Defined as requirement to keep seed for 8 weeks at 4 °C before being able bolt or flower. ^d^ W—Top Heavy branching often seen with secondary branching; X—Branched length of main stem with few secondary branches; Y—Tiller where all branches originate from the base and contain few secondary branching; Z—Chaos where branches are seen originating everywhere and a dominant stem was not observed.^e^ Defined as time when greater than 50% of all floret within the initial composite flower are open. ^f^ Maximum height of plant as determined at initial/primary flowering. ^g^ Number of florets seen in the initial composite flower on the initial floral bolt. ^h^ Defined as time when greater than 50% of all flowers are open and seed set has begun. ^i^ Maximum height of plant at maturity.

**Table 9:** NGRP center designations.

Accession	Lot#	Scientific Name	Other Name(s)	Country of Origin	Donor
PI 258366	06ncai01	*Camelina sativa*	VNIIMK 17	Soviet Union	NGRP
PI 258367	06ncai01	*Camelina sativa*	Voronezh 349	Soviet Union	NGRP
PI 304269	06ncai02	*Camelina sativa*	No. 402	Sweden	NGRP
PI 304270	97ncei01	*Camelina sativa*	No. 403	Sweden	NGRP
PI 304271	06ncai02	*Camelina sativa*	No. 406	Sweden	NGRP
PI 311735	06ncai01	*Camelina sativa*	Ames 1043	Poland	NGRP
PI 311736 *	94ncai01	*Camelina sativa*	Ames 1042	Poland	NGRP
PI 597833A	94ncai01	*Camelina sativa*	Ames 21330; 163-2073-72	Denmark	NGRP
PI 597833B	94ncai01	*Camelina sativa*	Ames 21330; 163-2073-72	Denmark	NGRP
PI 633192	97ncai01	*Camelina sativa*	Ames 22964; CR476/65; Pernice	Germany	NGRP
PI 633193 *	97ncai01	*Camelina sativa*	Ames 22985; CR492/94a	Germany	NGRP
PI 633194	97ncai01	*Camelina sativa*	Ames 22987; CR 1674/90; Giessen Nr. 3	Germany	NGRP
PI 650140	97ncai01	*Camelina sativa*	Ames 22986; CR 1673/90d; Came	Germany	NGRP
PI 650141	04ncai01	*Camelina sativa*	Ames 24253; NU 52279;	United States, Minnesota	NGRP
PI 650142A	02ncai01	*Camelina sativa*	Ames 26665; G 31712; CS-163-2073-72	Denmark	NGRP
PI 650142B	02ncai01	*Camelina sativa*	Ames 26665; G 31712; CS-163-2073-72	Denmark	NGRP
PI 650143 *	06ncai01	*Camelina sativa*	Ames 26666; G 31713; CS-CR00	Germany	NGRP
PI 650144	02ncni01	*Camelina sativa*	Ames 26667; G 31714; CS-CR1670; Boha	Denmark	NGRP
PI 650145	06ncai01	*Camelina sativa*	Ames 26668; G 31715; CS-CR1671; BRSCHW 28347	Germany, Mecklenburg-W.P.	NGRP
PI 650146	06ncai01	*Camelina sativa*	Ames 26669; G 31716; CS-CR1672; BRSCHW 30021	Sweden	NGRP
PI 650147	02ncni01	*Camelina sativa*	Ames 26670; G 31717; CD-CR1673d; Came	Sweden	NGRP
PI 650148	02ncai01	*Camelina sativa*	Ames 26671; G 31718; CS-CR1674; Giessen #3	Germany, Mecklenburg-W.P.	NGRP
PI 650149	02ncai01	*Camelina sativa*	Ames 26672; G 31719; CS-CR1675; Giessen #4	Germany, Mecklenburg-W.P.	NGRP
PI 650150	02ncai01	*Camelina sativa*	Ames 26673; G 31720; CS-CR1676; Hoga	Denmark	NGRP
PI 650151	02ncai01	*Camelina sativa*	Ames 26674; G 31721; CS-CR1677; Svalof	Sweden	NGRP
PI 650152 *	08ncai01	*Camelina sp*	Ames 26675; G 31722; CPS-CAM23	Germany	NGRP
PI 650153	02ncai01	*Camelina sativa*	Ames 26676; G 31723; CPS-CAM10	Soviet Union	NGRP
PI 650154	02ncni01	*Camelina sativa*	Ames 26677; CSS-CAM25; G 31724;	Soviet Union	NGRP
PI 650155*	02ncai01	*Camelina sativa*	Ames 26678; CSS-CAM27; G 31725;	Poland	NGRP
PI 650156	02ncni01	*Camelina sativa*	Ames 26679; CSS-CAM29; G 31726	Soviet Union	NGRP
PI 650157A *	02ncai01	*Camelina sativa*	Ames 26680; CSS-CAM30; G 31727	Soviet Union	NGRP
PI 650157B	02ncai01	*Camelina sativa*	Ames 26680; CSS-CAM30; G 31727;	Soviet Union	NGRP
PI 650158A *	02ncai01	*Camelina sativa*	Ames 26681; CSS-CAM31; G 31728	Poland	NGRP
PI 650158B	02ncai01	*Camelina sativa*	Ames 26681; CSS-CAM31; G 31728;	Poland	NGRP
PI 650159	02ncni01	*Camelina sativa*	Ames 26662; CSS-CAM33; G 31729	Poland	NGRP
PI 650163	02ncni01	*Camelina sativa*	Ames 26686; CSS-CAM37; G 31733	Soviet Union	NGRP
PI 650164	02ncni01	*Camelina sativa*	Ames 26687; CSS-CAM38; G 31734	Austria	NGRP
PI 650165	02ncni01	*Camelina sativa*	Ames 26688; CSS-CAM7; G 37135	Soviet Union	NGRP
PI 650166	02ncni01	*Camelina sativa*	Ames 26689; CSS-CAM8; G 31736	Soviet Union	NGRP
PI 650167 *	08ncai01	*Camelina sativa*	Ames 27286; Index Seminum 144	Poland, Przemysl	NGRP
PI 650168 *	08ncai01	*Camelina sativa*	Ames 28372; NE2006-01	United States, Nebraska	NGRP
Suneson ^a^		*Camelina sativa*	Ames 1043; Ames 26665; "Calena" A3U7761	United States, Montana	U of Mont

* Indicates accessions that required vernalization in order to flower. Also described as ‘winter’ accessions. A and B designation were given to accession obtained from the NGRP center that contained more than two unique phenotypes. Accession PI 650152 was designated *Camelina sp.* due to its unique chromosome count described later. ^a^ Suneson is a commonly used accession in the US not provided by NGRP. Suneson or Montana 0305 was release by Montana State University as a mid-season, average-yield line that is high in α-linolenic acid (C18:3n3). It was included in this study for its known ability to be transformed by the floral dip method.

## Supplementary Materials

The following are available online at https://www.mdpi.com/2223-7747/9/5/642/s1, Figure S1. Selectable markers vectors for *Camelina*; Figure S2. *npt*II marker assessment for *Camelina*; Figure S3. *hpt*II marker assessment for *Camelina*; Figure S4. *sul*I marker assessment for *Camelina*; Figure S6. 5-fluorocytosine (5FC) kill curve analysis of *Camelina*; Figure S7. PCR analysis of putative transgenic PI 311735 and Suneson Camelina; Table S1. Raw Data Oil Analysis; Table S2. Raw Data Compilation Supplemental; Table S3. TSW Stats; Table S4. Total seed weight Stats.
