# Supplementary material for: Phenotypic Examination of Camelina sativa (L.) Crantz Accessions from the USDA-ARS National Genetics Resource Program
Source: Plants (Basel). 2020 May 19;9(5):642. doi: 10.3390/plants9050642 (PMC7286027; doi:10.3390/plants9050642)
Supplement: Supplementary file 1 [file plants-09-00642-s001.zip › 042620 Camelina Figures S1-S7.pptx]

## Slide 1
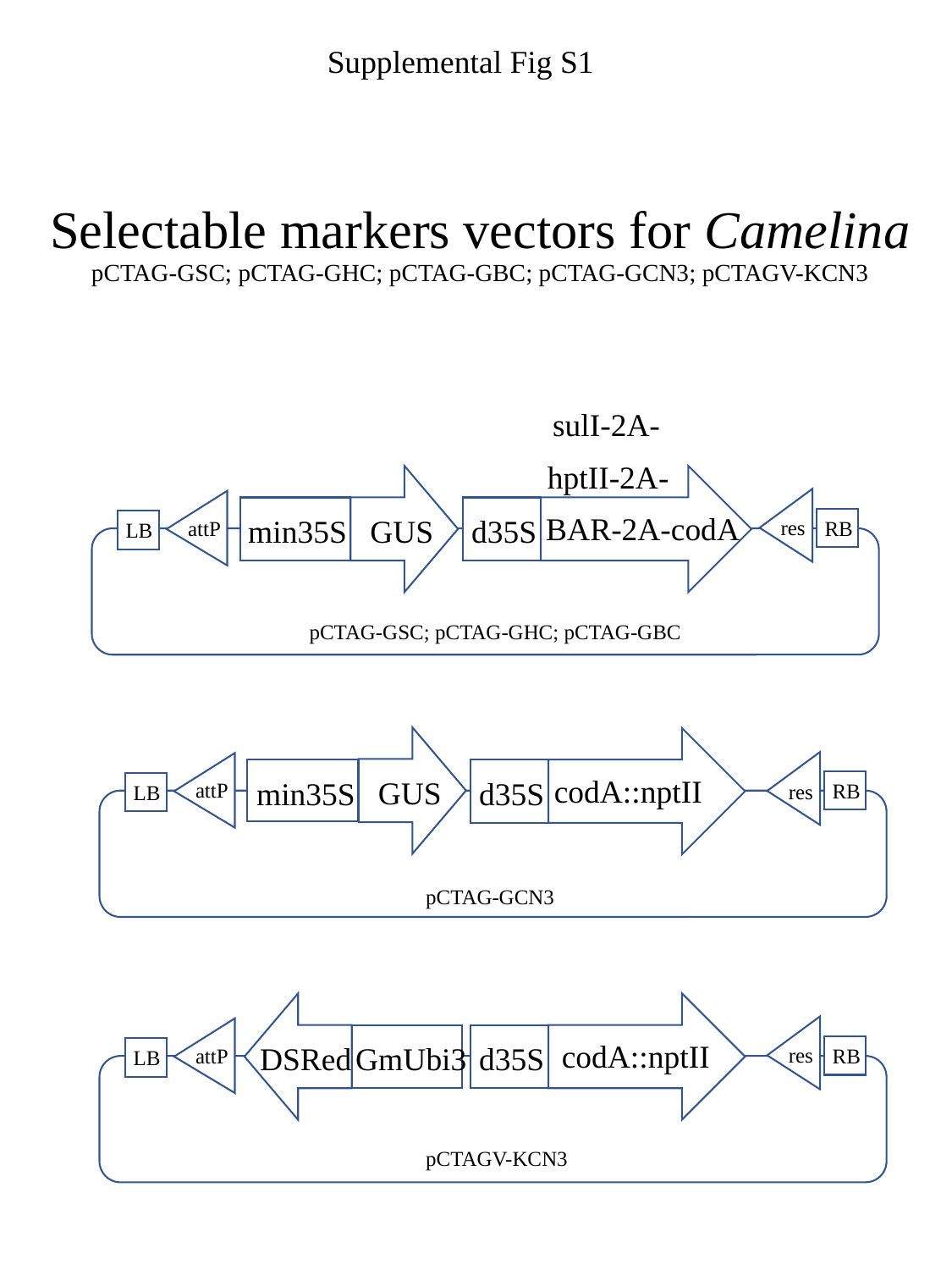

Supplemental Fig S1
# Selectable markers vectors for Camelina pCTAG-GSC; pCTAG-GHC; pCTAG-GBC; pCTAG-GCN3; pCTAGV-KCN3
sulI-2A-
hptII-2A-
BAR-2A-codA
res
attP
min35S
d35S
GUS
RB
LB
pCTAG-GSC; pCTAG-GHC; pCTAG-GBC
codA::nptII
res
attP
min35S
d35S
GUS
RB
LB
pCTAG-GCN3
codA::nptII
res
attP
GmUbi3
d35S
DSRed
RB
LB
pCTAGV-KCN3

## Slide 2
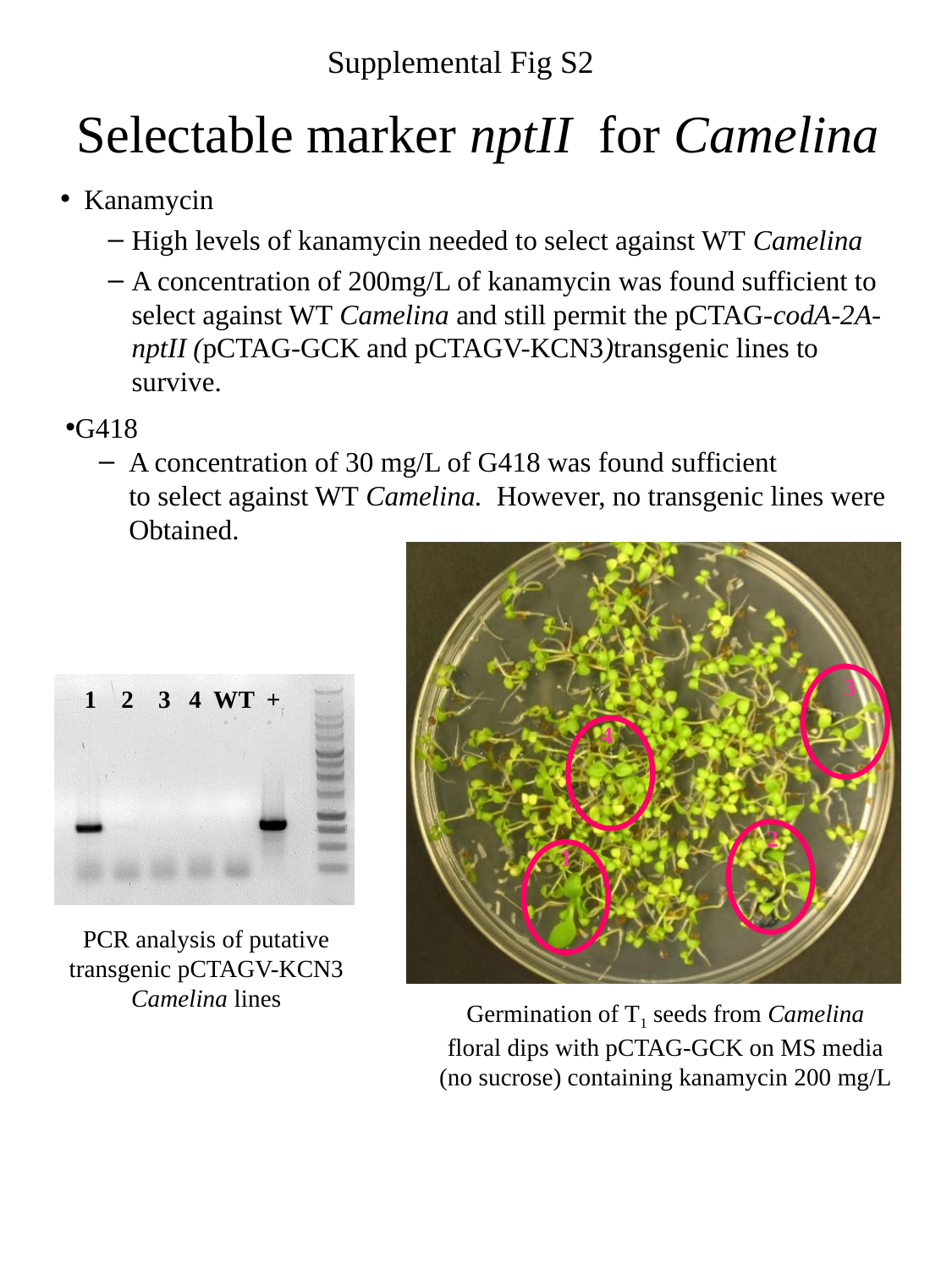

Supplemental Fig S2
# Selectable marker nptII for Camelina
Kanamycin
High levels of kanamycin needed to select against WT Camelina
A concentration of 200mg/L of kanamycin was found sufficient to select against WT Camelina and still permit the pCTAG-codA-2A-nptII (pCTAG-GCK and pCTAGV-KCN3)transgenic lines to survive.
G418
A concentration of 30 mg/L of G418 was found sufficient
to select against WT Camelina. However, no transgenic lines were
Obtained.
3
 1 2 3 4 WT +
4
2
1
PCR analysis of putative transgenic pCTAGV-KCN3 Camelina lines
Germination of T1 seeds from Camelina floral dips with pCTAG-GCK on MS media (no sucrose) containing kanamycin 200 mg/L

## Slide 3
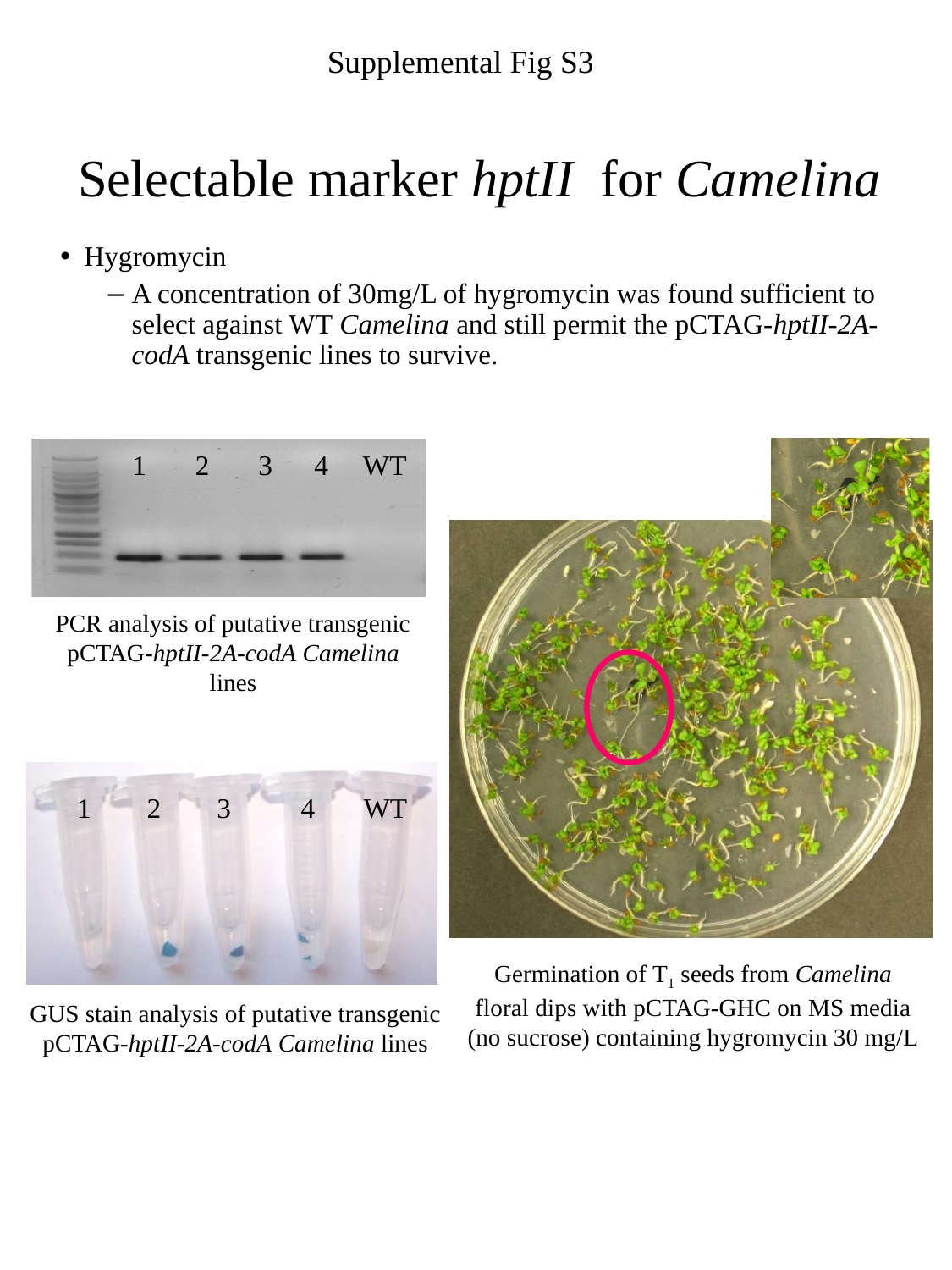

Supplemental Fig S3
# Selectable marker hptII for Camelina
Hygromycin
A concentration of 30mg/L of hygromycin was found sufficient to select against WT Camelina and still permit the pCTAG-hptII-2A-codA transgenic lines to survive.
1 2 3 4 WT
PCR analysis of putative transgenic pCTAG-hptII-2A-codA Camelina lines
1 2 3 4 WT
Germination of T1 seeds from Camelina floral dips with pCTAG-GHC on MS media (no sucrose) containing hygromycin 30 mg/L
GUS stain analysis of putative transgenic pCTAG-hptII-2A-codA Camelina lines

## Slide 4
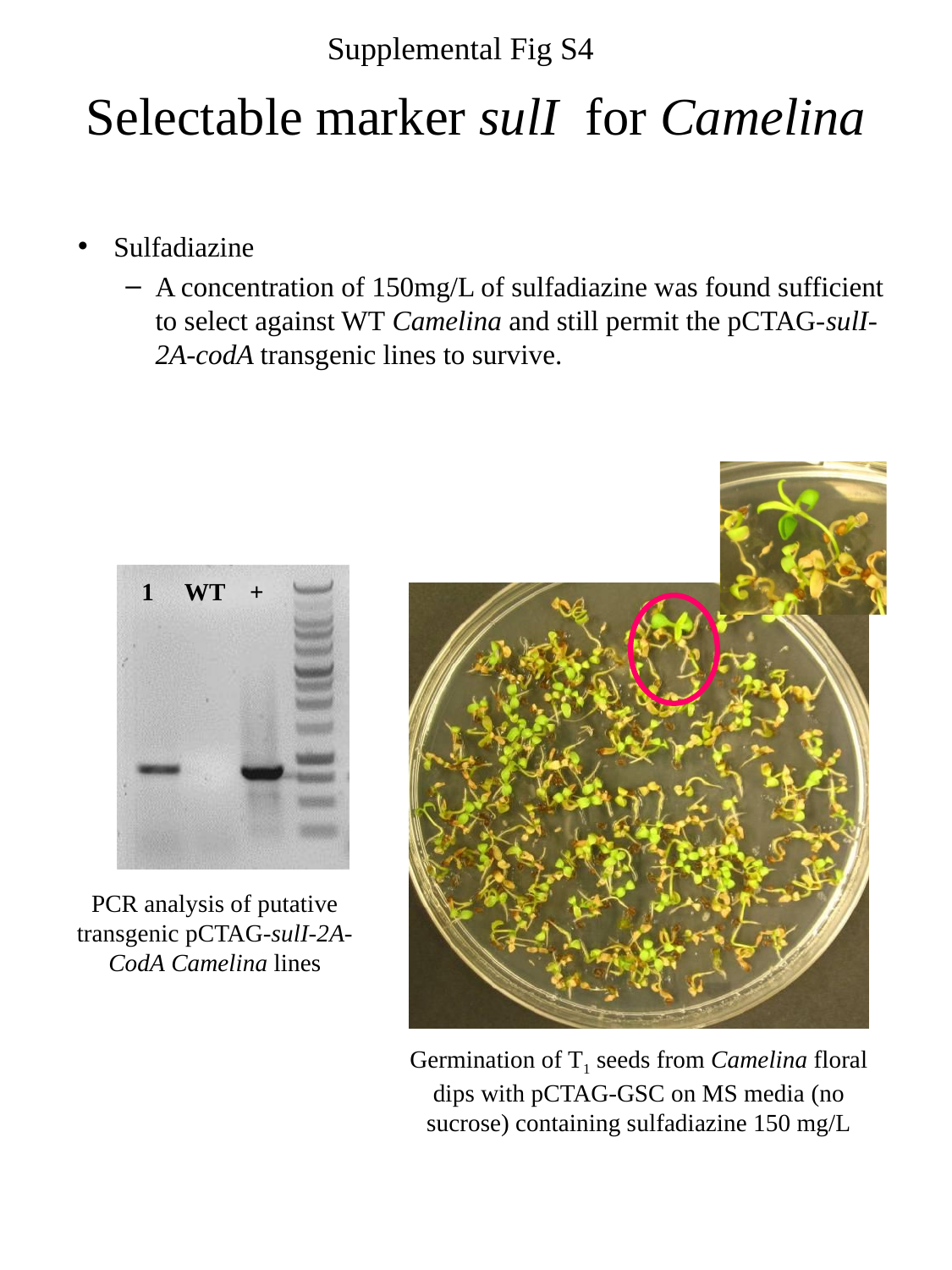

Supplemental Fig S4
# Selectable marker sulI for Camelina
Sulfadiazine
A concentration of 150mg/L of sulfadiazine was found sufficient to select against WT Camelina and still permit the pCTAG-sulI-2A-codA transgenic lines to survive.
1 WT +
PCR analysis of putative transgenic pCTAG-sulI-2A-CodA Camelina lines
Germination of T1 seeds from Camelina floral dips with pCTAG-GSC on MS media (no sucrose) containing sulfadiazine 150 mg/L

## Slide 5
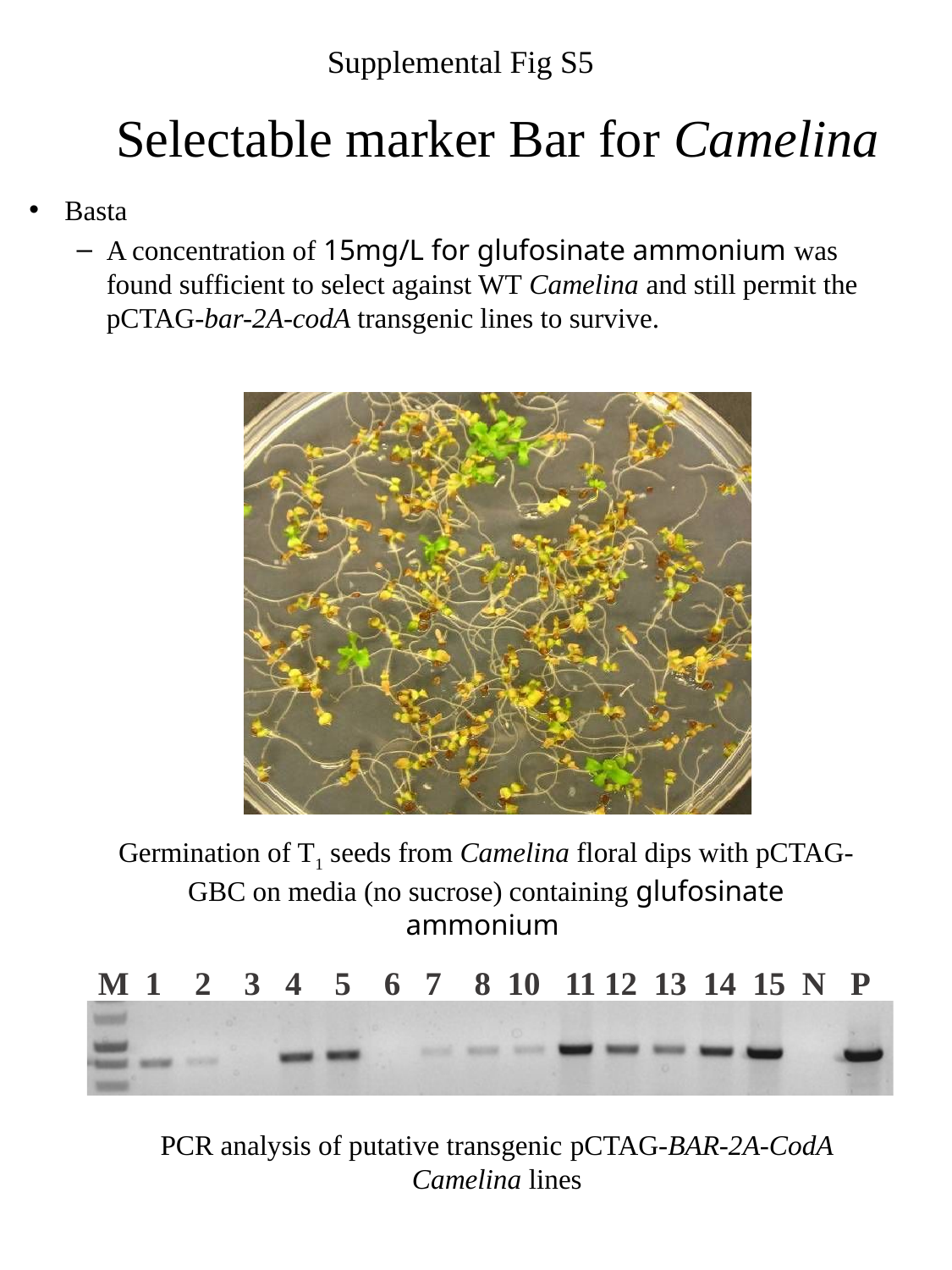

Supplemental Fig S5
Selectable marker Bar for Camelina
Basta
A concentration of 15mg/L for glufosinate ammonium was found sufficient to select against WT Camelina and still permit the pCTAG-bar-2A-codA transgenic lines to survive.
Germination of T1 seeds from Camelina floral dips with pCTAG-GBC on media (no sucrose) containing glufosinate ammonium
 M 1 2 3 4 5 6 7 8 10 11 12 13 14 15 N P
PCR analysis of putative transgenic pCTAG-BAR-2A-CodA Camelina lines

## Slide 6
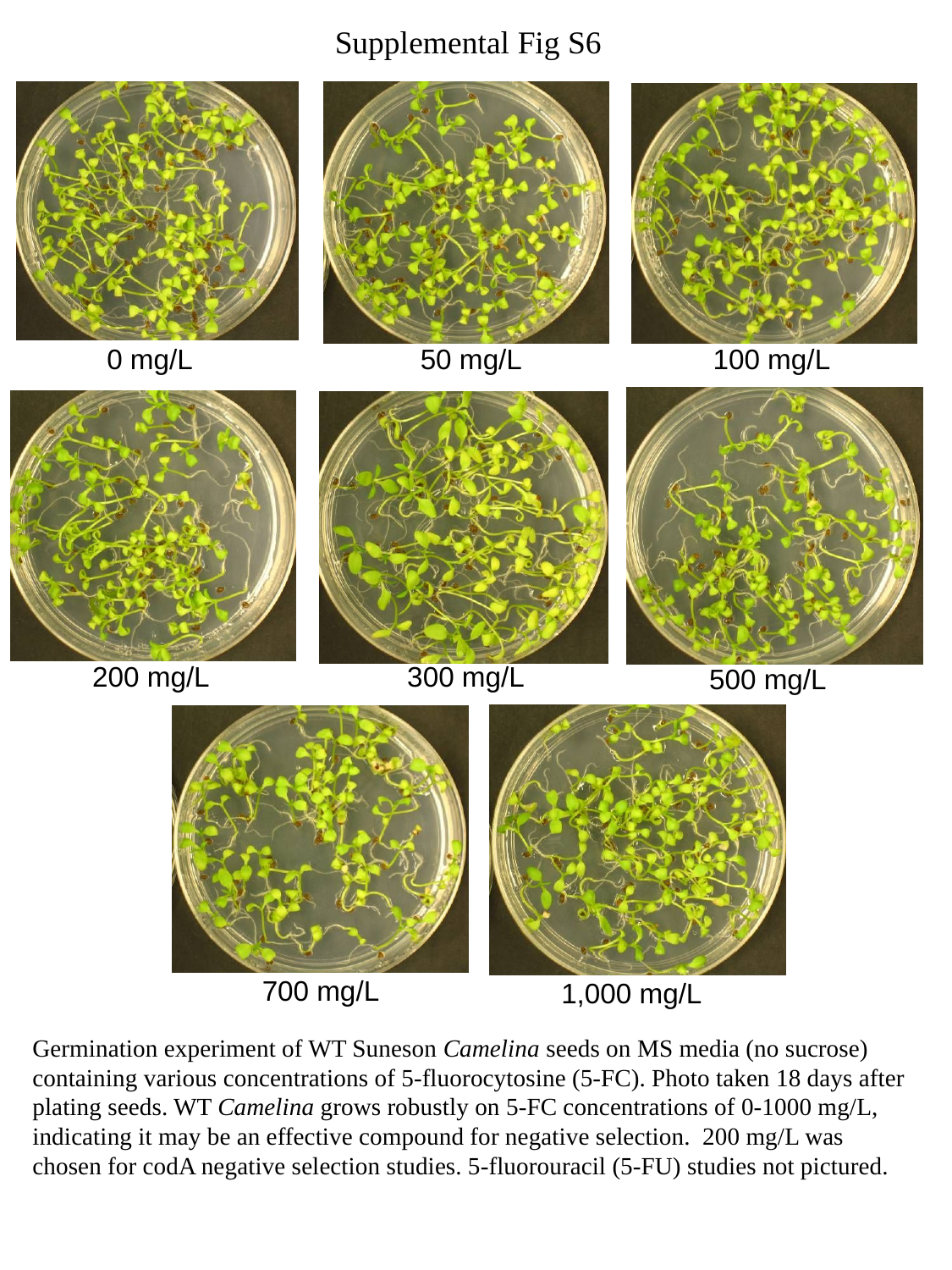

Supplemental Fig S6
0 mg/L
50 mg/L
100 mg/L
200 mg/L
300 mg/L
500 mg/L
700 mg/L
1,000 mg/L
Germination experiment of WT Suneson Camelina seeds on MS media (no sucrose) containing various concentrations of 5-fluorocytosine (5-FC). Photo taken 18 days after plating seeds. WT Camelina grows robustly on 5-FC concentrations of 0-1000 mg/L, indicating it may be an effective compound for negative selection. 200 mg/L was chosen for codA negative selection studies. 5-fluorouracil (5-FU) studies not pictured.

## Slide 7
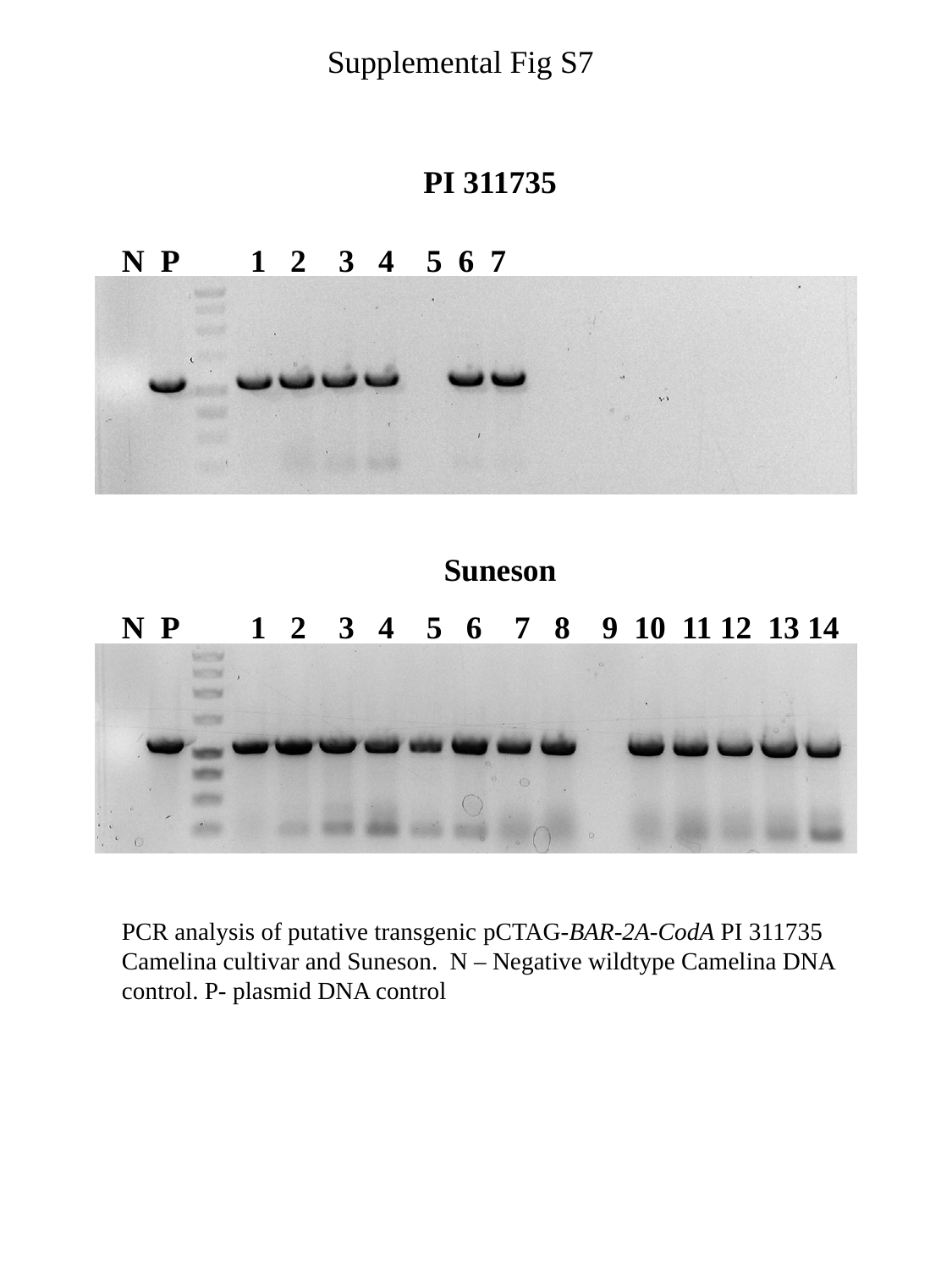

Supplemental Fig S7
PI 311735
N P 1 2 3 4 5 6 7
Suneson
N P 1 2 3 4 5 6 7 8 9 10 11 12 13 14
PCR analysis of putative transgenic pCTAG-BAR-2A-CodA PI 311735 Camelina cultivar and Suneson. N – Negative wildtype Camelina DNA control. P- plasmid DNA control
